# Association of Left Angular Gyrus‐Right Middle Frontal Gyrus Functional Connectivity With Insomnia Severity in Major Depressive Disorder: Evidence From the DIRECT Consortium

**DOI:** 10.1002/cns.71083

**Published:** 2026-08-13

**Authors:** Zhifang Zhang, Zhenzhu Chen, Yimeng Wang, Lei Zhao, Feng Li, Yujie Xing, Tian Li, Qi‐Jing Bo, Chuanyue Wang

**Affiliations:** ^1^ Beijing Key Laboratory of Intelligent Drug Research and Development for Mental Disorders; National Clinical Research Center for Mental Disorders; National Center for Mental Disorders; Beijing Anding Hospital Capital Medical University Beijing China; ^2^ Beijing Institute for Brain Disorders Center of Schizophrenia, Beijing Anding Hospital Capital Medical University Beijing China; ^3^ Advanced Innovation Center for Human Brain Protection Capital Medical University Beijing China

**Keywords:** functional connectivity, functional magnetic resonance imaging, insomnia severity, local brain function, major depressive disorder

## Abstract

**Background:**

Identifying neuroimaging correlates of insomnia severity could provide insights into the underlying biological mechanisms of major depressive disorder (MDD). However, related findings remain inconsistent, and the functional connectivity patterns associated with insomnia severity are unclear.

**Methods:**

This study analyzed resting‐state fMRI data from 385 patients with MDD and 336 healthy controls (HCs) sourced from nine sites of the DIRECT Consortium. Patients were stratified into high insomnia (MDDHI; HAMD insomnia subscale ≥ 4, *n* = 226) and low insomnia (MDDLI; HAMD insomnia subscale ≤ 3, *n* = 159) groups. Among patients with MDDHI, MDDLI and HCs, we first examined network‐level functional connectivity abnormalities using the Craddock 200 atlas, and then local brain function was assessed using the amplitude of low‐frequency fluctuations (ALFF), regional homogeneity (ReHo) and degree centrality (DC). Finally, we adopted multiple analytical approaches to verify the robustness of the significant findings.

**Results:**

Compared to both patients with MDDHI and HCs, patients with MDDLI exhibited significantly reduced functional connectivity between the left angular gyrus (AG) and the right middle frontal gyrus (MFG). Both patients with MDDHI and MDDLI showed significantly decreased ALFF and ReHo values relative to HCs across multiple brain regions, including bilateral angular gyrus/precuneus/cerebellum posterior lobe and so on. For DC, patients with MDDHI showed significantly decreased values relative to HCs in all identified clusters, whereas patients with MDDLI showed significant DC reductions in a subset of these clusters. When these results were validated using multiple analytical approaches, the primary findings remained consistent.

**Conclusions:**

Reduced functional connectivity between the left AG and the right MFG may be a candidate neuroimaging marker for sleep‐related heterogeneity in MDD. However, widespread local brain function abnormalities may reflect core depressive pathology of MDD. These findings advance our understanding of the neurobiology of MDD.

## Graphical abstract

Preferential alterations in insomnia‐related AG–MFG connectivity in MDD highlight sleep‐associated neural mechanisms, although disentangling these connectivity changes from depression severity remains challenging. Local aberrations in regional brain function primarily differentiate patients with MDD from healthy controls.

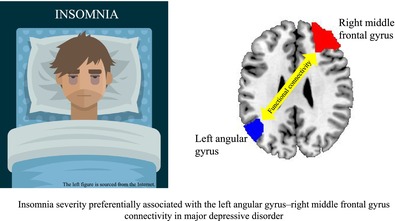

## 1 Introduction

Major depressive disorder (MDD) is a chronic and debilitating mental illness characterized by persistent sadness, anhedonia, and sleep disturbances [1]. Previous studies have found that insomnia in young adults was associated with significantly increased risk of major depressive episodes [2]. Moreover, insomnia frequently co‐occurs with MDD and often persists even during remission [3, 4]. Insomnia in MDD is associated with increased suicide risk, poorer social functioning [5], worse treatment outcomes [6], is considered an independent contributor to MDD etiology [7], a strong predictor of relapse [8], and shows evidence of a causal relationship with MDD [9].

Neuroimaging studies have suggested overlapping neural mechanisms between MDD and insomnia [10, 11, 12, 13]. For instance, functional connectivity involving the lateral orbitofrontal cortex, precuneus, and angular gyrus has been linked to both depressive symptoms and sleep problems [14, 15]. As far as we know, six studies have specifically investigated brain functional abnormalities related to insomnia severity in MDD using various metrics. Two studies focused on abnormal functional interactions between different brain regions and found that, compared with patients with MDD who were with high insomnia (MDDHI), patients with low insomnia (MDDLI) exhibited decreased global functional connectivity density (gFCD) in the left inferior temporal gyrus, increased gFCD in bilateral parahippocampal/hippocampal gyri [16], decreased functional connectivity of the amygdala with bilateral superior temporal gyrus, and increased functional connectivity of the amygdala with the left supplementary motor area and bilateral postcentral gyrus [17]. The other four studies focused on abnormal local brain function of patients. Two studies found that, compared with patients with MDDHI, patients with MDDLI exhibited decreased static amplitude of low‐frequency fluctuations (ALFF) in the right inferior frontal gyrus/anterior insula [18], and increased dynamic ALFF in the right middle temporal gyrus/superior temporal gyrus [19]. The other two studies used regional homogeneity (ReHo) as index, but the results were inconsistent. One study found that, compared with patients with MDDHI, patients with MDDLI showed decreased ReHo values in the right middle temporal gyrus, and increased ReHo values in the left orbital superior frontal gyrus, the lingual gyrus, the right inferior parietal gyrus and the postcentral gyrus [20]. However, the other study found decreased ReHo values of patients with MDDLI in the right posterior cingulate the cortex/precuneus, the right median cingulate cortex, the left postcentral gyrus, and the right inferior temporal gyrus [21]. These inconsistencies may stem from methodological limitations, including small sample sizes (22–33 per group), varying gender ratios, single‐center designs, and potential sampling bias. Critically, no large‐scale, multi‐center study has yet identified robust neurobiological correlates of insomnia severity within MDD, particularly from a whole‐brain network perspective in adults. Identifying such correlates could pave the way for personalized therapies targeting insomnia in severe depression.

This study aimed to identify local and network‐level functional abnormalities associated with insomnia severity in MDD using multi‐center, large‐sample data. First, based on the Craddock et al. 200‐region brain parcellation (i.e., the CC200 atlas) [22], a 200 × 200 functional matrix was constructed to explore whole‐brain functional connectivity abnormalities. Second, local brain function abnormalities were examined using the ALFF, ReHo and degree centrality (DC). Third, validation analyses were employed to confirm the robustness of significant findings. It is expected that our results would expand our understanding of MDD pathophysiology and move toward identifying symptom‐specific biological subtypes.

## 2 Materials and Methods

### 2.1 Subjects

This study recruited 1300 patients with MDD and 1128 healthy controls. All of them were part of the DIRECT consortium [23, 24]. All patients met the diagnostic criteria of MDD according to the Diagnostic and Statistical Manual of Mental Disorders, Fourth Edition (DSM‐IV) or the International Classification of Diseases 10 (ICD‐10) criteria for MDD. The sample selection process was detailed in Figure S1. Demographic and clinical characteristics of the final sample were presented in Table 1. Sample sizes per site were shown in Table S1. Demographic data and group differences by site were presented in Table S2. The severity of patients' depressive symptoms were assessed using the 17‐item Hamilton Depression Rating Scale (HAMD) [25]. Site differences in HAMD total scores were shown in Table S3. Following the practice of previous researches [17, 18, 26], insomnia severity was evaluated using the HAMD insomnia subscale (items 4 [early], 5 [middle], and 6 [late] insomnia). In the present sample, the internal consistency of this 3‐item subscale was acceptable (Cronbach's α = 0.626; site‐stratified values were provided in Table S4). Patients were classified as MDDLI (subscale total ≤ 3) or MDDHI (subscale total ≥ 4). The ≥ 4 threshold operationalizes a conservative but clinically meaningful construct: it identifies patients with either multi‐domain mild insomnia or single‐domain moderate‐to‐severe insomnia with additional mild involvement (e.g., 2 + 1 + 1 = 4). This aligns with the clinical concept of “high insomnia burden” requiring targeted intervention, as opposed to patients with isolated mild symptoms who may represent a distinct phenotypic subgroup. To verify the rationality of the grouping cut‐off value, a receiver operating characteristic (ROC) curve analysis was conducted using an internal criterion (any HAMD insomnia subscale item ≥ 2) [27]. This analysis yielded an Area Under Curve (AUC) of 95.7% (95% CI: 94.0%–97.4%), with sensitivity of 79.6% and specificity of 100.0% at the ≥ 4 threshold (Supporting Information). In accordance with ethics standards, the study was approved by each site's ethics committee, and written informed consent was secured from every participant before formal testing.

**TABLE 1:** Demographic and clinical characteristics of patients with Major Depressive Disorder who were with high/low insomnia and healthy controls.

	MDDHI	MDDLI	HCs	H/Z/χ^2^(*p*)
Age (Median (Q1, Q3))^a^	32.50 (26,43)	30 (24,39)	30 (24,42)	6.85 (0.033*)
Gender (male/female)^b^	87/139	63/96	153/183	3.23 (0.199)
Education level (Median (Q1, Q3))^a^	12 (9,15)	13 (9,15)	15 (11,15)	30.23 (< 0.001*)
Mean FD (Median (Q1, Q3))^a^	0.05 (0.04,0.08)	0.06 (0.04,0.09)	0.06 (0.04,0.08)	1.96 (0.376)
Adjusted HAMD total score (Median (Q1, Q3))^c^	18 (16,23)	16 (10,19)	—	−6.09 (< 0.001*)

*Note:* MDDHI: Patients with major depressive disorder who were with high insomnia; MDDLI: Patients with major depressive disorder who were with low insomnia.

Abbreviations: FD: Framewise displacement; HAMD: Hamilton depression scale; HCs: Healthy controls.

^a^ These variables were compared by using Kruskal‐Wallis test.

^b^ This variable was compared by using χ^2^ test.

^c^ This variable was compared by using Mann–Whitney U test; The adjusted HAMD total score did not include the scores of items 4, 5, and 6 in the calculation.

* *p* < 0.05.

Medication status at enrollment was available for 262 of the 385 patients (missing: 123, 32.0%). Among these, 174 were patients with MDDHI (87 medicated, 87 unmedicated) and 88 were patients with MDDLI (39 medicated, 49 unmedicated). However, detailed information on drug type and dosage was not known for any participant.

### 2.2 Imaging Data Acquisition and Preprocessing

Resting‐state fMRI and T1‐weighted structural data were acquired at each site using various MRI scanners and key parameters were shown in Table S1. All imaging data were preprocessed in the DPABI software (https://rfmri.org/DPABI) by using the same DPARSF protocol [23]. Briefly, the preprocessing steps included discarding the first ten time points, slice‐timing correction, realignment to the first image to correct for head motion, normalization to the Montreal Neurological Institute (MNI) space (T1 images were used, resampling voxel size = 3 × 3 × 3 mm^3^), nuisance covariate regression (including Friston‐24 parameters for head motion, white matter signal, cerebrospinal fluid signal, global signal, and linear trend term for detrending), temporal bandpass filtering (0.01–0.10 Hz). Notably, volume censoring, frame‐wise scrubbing and spatial smoothing were omitted throughout preprocessing. Preprocessed data entered subsequent data analyses.

### 2.3 Construction of Functional Connectivity Matrix

Functional brain networks were constructed in the DPABI software. After preprocessing, time series of each voxel within each region of interest (ROI) of the CC200 atlas [22] were extracted and averaged. ROIs in the CC200 atlas encompassed all voxels in the brain's gray matter including cerebral and subcortex, midbrain and cerebellum. Then, Pearson correlation analysis was performed to estimate functional connectivity between all pairs of ROIs across all subjects. Finally, Fisher's r‐to‐z transformation was applied to the 200 * 200 functional connectivity matrices. Each subject's functional connectivity matrices entered the second‐level analysis. It needs to be mentioned that all the functional connectivity matrices were not sparsified (i.e., thresholded), as the primary analysis involved connection‐wise group comparisons of continuous connectivity strengths rather than graph‐theoretical network analyses. Noise was minimized through rigorous preprocessing (see 2.2), and false positives were controlled via FDR correction at the group level (see 2.5).

### 2.4 Calculation of ALFF, ReHo and DC

ALFF, ReHo, and DC were calculated in DPABI as described previously [23]. ALFF reflects the strength or intensity of low frequency oscillations [28], ReHo measures the temporal synchronization between a given voxel and its 26 nearest neighbors (here, 27 voxels) [29], and DC quantifies the importance of a given voxel in the whole‐brain network by counting its functional connections (weighted DC, *r* > 0.25) [30, 31].

### 2.5 Statistical Analyses

Differences among patients with MDDHI, MDDLI, and HCs in age, gender, education level, and mean framewise displacement (FD) were assessed using Kruskal‐Wallis, χ^2^, or Mann–Whitney U tests in SPSS 26.0 (SPSS Inc., Chicago, IL, USA). Post hoc analyses used Bonferroni correction (*p* < 0.05).

Group differences (patients with MDDHI vs. patients with MDDLI vs. HCs) in functional connectivity were assessed using one‐way ANCOVA in DPABI. Significant level was set at the FDR–corrected *p* value (*P*_FDR‐corrected_) of < 0.05. For significant connections, the values of each connection were extracted, and post hoc analyses (Bonferroni corrected, *p* < 0.05) were performed in SPSS 26.0, respectively.

Second‐level analyses of ALFF, ReHo and DC were conducted in the SPM12 software (Wellcome Department of Cognitive Neurology, London, UK), respectively. In these analyses, one‐way ANCOVA was used. The independent variable was group (i.e., patients with MDDHI vs. patients with MDDLI vs. HCs). Voxel‐level significance was initially assessed at an uncorrected threshold of *p* < 0.001 to define contiguous clusters. Cluster‐level family‐wise error (FWE) correction was then applied using Gaussian random field theory as implemented in SPM12. Clusters surviving *P*_FWE_ < 0.05 were considered statistically significant. The smoothness of the residuals was estimated from the data, and the search volume was restricted to a pre‐made gray matter mask (intersection of a whole‐brain mask covering ≥ 90% of subjects from the DIRECT consortium and the DPABI gray mask). Once a significant cluster was found, the ALFF, ReHo or DC value of each voxel was extracted and averaged for each subject. Then, in the SPSS 26.0, post hoc analyses were performed and Bonferroni correction was used (*p* < 0.05).

What's more, to control for potential confounding influences, all second‐level imaging data analyses included age, gender, education level, mean FD, and scanning site as covariates of no interest. This ensures that any observed group effects were statistically adjusted for demographic and motion‐related variance.

Furthermore, considering the abnormal functional connectivity of the left angular gyrus with the right middle frontal gyrus (AG‐MFG) was the primary novel finding, we further conducted a correlation analysis between the AG‐MFG functional connectivity and the total score of the HAMD insomnia subscale. We first examined the normality of the data using the Kolmogorov–Smirnov test. The results indicated that the total score of the HAMD insomnia subscale did not follow a normal distribution, either in the whole sample or in the MDDHI and MDDLI subgroups. Therefore, we employed Spearman's correlation analysis to assess the relationship between the AG‐MFG functional connectivity and the total score of the HAMD insomnia subscale.

### 2.6 Validation Analyses

To address the debated impact of global signal regression [32], all primary analyses (functional connectivity, ALFF, ReHo, DC) were repeated using data without global signal regression. Furthermore, as abnormal functional connectivity of the AG‐MFG was the primary novel finding, four complementary sensitivity analyses were performed to assess the robustness of this result. First, a leave‐one‐site‐out cross‐validation was performed to assess the influence of any single site. This analysis was implemented by repeating the second‐level analysis while excluding one site at a time. Second, after applying ComBat harmonization to the functional connectivity matrices, the second‐level analysis was repeated. Third, medication status (unmedicated, medicated, or unknown) was included as an additional covariate in the second‐level analysis of functional connectivity. Fourth, the second‐level analysis was repeated using only unmedicated patients. Fifth, a partial correlation analysis was conducted between the AG‐MFG connectivity and the HAMD insomnia subscale score, controlling for adjusted HAMD total score (i.e., total HAMD score minus the insomnia subscale), age, gender, education, mean FD, and scanning site, restricted to the patient sample (MDDHI and MDDLI).

## 3 Results

### 3.1 Demographic and Clinical Factor Analyses

Significant group differences were found among patients with MDDHI, MDDLI, and HCs in age (*p* = 0.033) and education level (*p* < 0.001) (Table 1). Further post hoc analyses showed that patients with MDDHI were significantly older than patients with MDDLI (*p* = 0.029), and both patients with MDDHI and MDDLI had lower education levels than HCs (*p* ≤ 0.001). Besides, patients with MDDHI had higher HAMD total scores than patients with MDDLI (*p* < 0.001). No significant difference was found in other demographic and clinical factors (all *p* > 0.05).

### 3.2 Functional Connectivity Analyses

**FIGURE 1:**
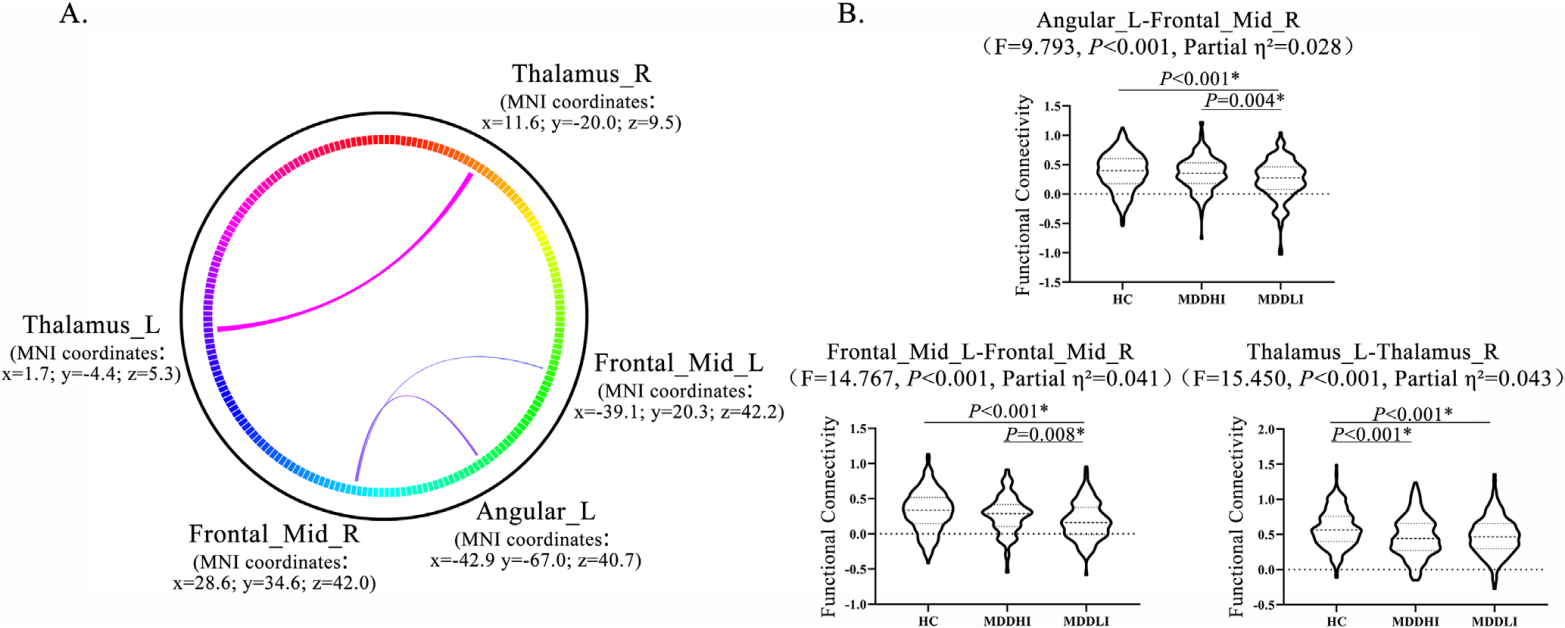
Functional connectivity differences among patients with MDD who were with high/low insomnia and HCs. The left part is the significant connections, and the right part is the violin picture of the averaged functional connectivity within each group.

Three connections showed significant group differences among patients with MDDHI, MDDLI, and HCs (Figure 1). They were functional connectivity of the AG–MFG and functional connectivity between the left and right middle frontal gyrus, and between the left and right thalamus. Further post hoc analyses showed that, for the first two connections, patients with MDDLI exhibited decreased functional connectivity than both patients with MDDHI and HCs; for the third connection, both patients with MDDHI and MDDLI exhibited decreased functional connectivity than HCs.

### 3.3 ALFF, ReHo And DC Analyses

**FIGURE 2:**
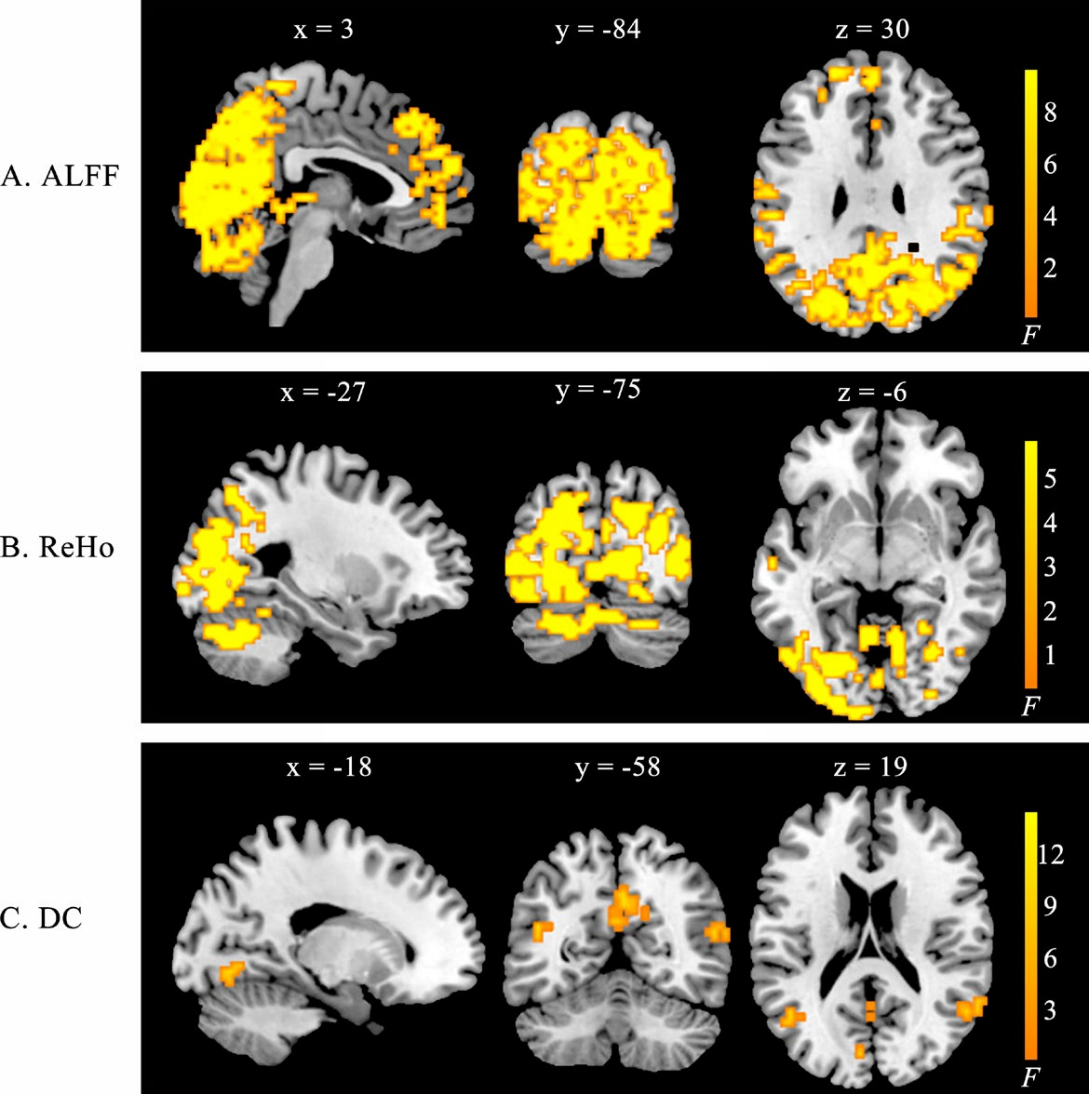
Significant brain regions identified in the ALFF, ReHo and DC analyses among patients with MDD who were with high/low insomnia and HCs.

For ALFF, 8 clusters showed significant group differences among patients with MDDHI, MDDLI, and HCs (Figure 2 and Table 2), including bilateral angular gyrus/precuneus/cerebellum posterior lobe/cuneus/lingual gyrus/middle temporal gyrus/middle occipital gyrus/limbic lobe, the left and right medial frontal gyrus, the left superior frontal gyrus, the left inferior parietal lobule, the left middle frontal gyrus, the left inferior frontal gyrus, the right superior temporal gyrus, and the left postcentral gyrus. Post hoc analyses showed that both patients with MDDHI and MDDLI exhibited decreased ALFF values compared to HCs in all clusters.

**TABLE 2:** Results of the ALFF, ReHo and DC analyses among patients with major depressive disorder who were with high/low insomnia and healthy controls.

	Brain region	Hemisphere	MNI coordinates	Cluster range coordinates	Peak F values	Cluster size	Cluster‐level *p*_FWE_	Post hoc analysis	
								Comparisons	*p*_Bonferroni_
ALFF	Angular/Precuneus/Cerebellum Posterior lobe/Cuneus/Lingual gyrus/Middle temporal g/Middle occipital gyrus/Limbic lobe	Left/Right	3, −84, 30	X: −63 to 66	23.58	8108	< 0.001*	MDDHI<MDDLI	1.000
				Y: −99 to −15				MDDHI<HC	< 0.001*
				Z: −39 to 69				MDDLI<HC	< 0.001*
	Medial Frontal Gyrus	Left/Right	0,57,21	X: −12 to 12	16.39	311	< 0.001*	MDDHI<MDDLI	1.000
				Y: 18 to 63				MDDHI<HC	< 0.001*
				Z: −18 to 51				MDDLI<HC	< 0.001*
	Superior Frontal Gyrus	Left	−21,54,30	X: −33 to −9	15.75	54	< 0.001*	MDDHI<MDDLI	1.000
				Y: 39 to 54				MDDHI<HC	< 0.001*
				Z: 15 to 33				MDDLI<HC	0.001*
			−21,33,45	X: −33 to −18	13.22	29	0.002*	MDDHI<MDDLI	1.000
				Y: 9 to 33				MDDHI<HC	< 0.001*
				Z: 39 to 51				MDDLI<HC	< 0.001*
	Inferior Parietal Lobule	Left	−45, −30, 45	X: −57 to −39	15.52	39	< 0.001*	MDDHI<MDDLI	1.000
				Y: −33 to −12				MDDHI<HC	0.001*
				Z: 33 to 51				MDDLI<HC	0.001*
	Middle Frontal Gyrus	Left	−21,63,12	X: −39 to −15	15.32	62	< 0.001*	MDDHI<MDDLI	1.000
				Y: 48 to 63				MDDHI<HC	< 0.001*
				Z: −3 to 18				MDDLI<HC	< 0.001*
	Inferior Frontal Gyrus	Left	−39,24,−21	X: −54 to −33	13.69	61	< 0.001*	MDDHI<MDDLI	1.000
				Y: 12 to 30				MDDHI<HC	< 0.001*
				Z: −27 to 0				MDDLI<HC	0.001*
			−45,24,15	X: −57 to −39	12.69	31	0.001*	MDDHI<MDDLI	1.000
				Y: 21 to 36				MDDHI<HC	< 0.001*
				Z: 9 to 24				MDDLI<HC	0.001*
			−57,12,15	X: −57 to −48	11.00	19	0.023*	MDDHI<MDDLI	1.000
				Y: 0 to 12				MDDHI<HC	0.001*
				Z: 12 to 24				MDDLI<HC	0.001*
	Superior Temporal Gyrus	Right	51,3,−9	X: 33 to 54	13.47	58	< 0.001*	MDDHI<MDDLI	1.000
				Y: −3 to 33				MDDHI<HC	< 0.001*
				Z: −21 to −6				MDDLI<HC	0.001*
			54, −15, 3	X: 48 to 63	11.87	28	0.002*	MDDHI<MDDLI	1.000
				Y: −24 to −9				MDDHI<HC	< 0.001*
				Z: −6 to 9				MDDLI<HC	0.001*
	Postcentral Gyrus	Left	−54, −3, 39	X: −63 to −48	10.36	30	0.001*	MDDHI<MDDLI	1.000
				Y: −18 to 0				MDDHI<HC	0.001*
				Z: 24 to 39				MDDLI<HC	0.001*
ReHo	Angular/Precuneus/Cuneus/Limbic Lobe/Cerebellum Posterior Lobe/Middle Occipital Gyrus/Middle Temporal Gyrus/Lingual Gyrus/Inferior Parietal Lobule	Left/Right	−27, −75, −6	X: −60 to 60	19.50	4260	< 0.001*	MDDHI<MDDLI	1.000
				Y: −102 to −24				MDDHI<HC	< 0.001*
				Z: −39 to 63				MDDLI<HC	< 0.001*
	Superior Temporal Gyrus	Left	−60, −21, 3	X: −66 to −57	14.82	62	< 0.001*	MDDHI<MDDLI	1.000
				Y: −27 to −12				MDDHI<HC	< 0.001*
				Z: −6 to 12				MDDLI<HC	< 0.001*
	Postcentral Gyrus	Left	−42, −27, 45	X: −54 to −39	14.28	74	< 0.001*	MDDHI<MDDLI	1.000
				Y: −30 to −12				MDDHI<HC	< 0.001*
				Z: 39 to 57				MDDLI<HC	< 0.001*
			−57, −12, 24	X: −66 to −57	10.83	22	0.041*	MDDHI<MDDLI	1.000
				Y: −15 to −9				MDDHI<HC	< 0.001*
				Z: 21 to 30				MDDLI<HC	0.002*
	Superior Parietal Lobule	Right	12, −54, 63	X: 9 to 27	13.60	58	< 0.001*	MDDHI<MDDLI	1.000
				Y: −57 to −45				MDDHI<HC	< 0.001*
				Z: 48 to 66				MDDLI<HC	< 0.001*
	Postcentral Gyrus	Right	63, −6, 27	X: 57 to 63	12.58	31	0.007*	MDDHI<MDDLI	1.000
				Y: −12 to 3				MDDHI<HC	< 0.001*
				Z: 12 to 30				MDDLI<HC	0.004*
DC	Superior Temporal Gyrus	Right	60, −54, 18	X: 51 to 63	13.72	17	0.011*	MDDHI<MDDLI	1.000
				Y: −60 to −51				MDDHI<HC	< 0.001*
				Z: 15 to 21				MDDLI<HC	< 0.001*
	Lingual	Left	−15, −72, −6	X: −21 to −12	13.17	16	0.015*	MDDHI<MDDLI	1.000
				Y: −81 to −66				MDDHI<HC	< 0.001*
				Z: −18 to −3				MDDLI<HC	0.229
	Precuneus	Left/Right	3, −60, 36	X: −9 to 15	12.70	85	< 0.001*	MDDHI<MDDLI	1.000
				Y: −75 to −51				MDDHI<HC	< 0.001*
				Z: 21 to 42				MDDLI<HC	< 0.001*
	Middle Temporal Gyrus	Left	−45, −57, 21	X: −48 to 39	11.43	15	0.022*	MDDHI<MDDLI	1.000
				Y: −66 to −54				MDDHI<HC	< 0.001*
				Z: 15 to 24				MDDLI<HC	< 0.001*
	Cuneus	Left	−9, −87, 30	X: −12 to −6	11.13	13	0.046*	MDDHI<MDDLI	0.706
				Y: −90 to −78				MDDHI<HC	< 0.001*
				Z: 18 to 30				MDDLI<HC	0.095

*Note:* MDDHI: Patients with major depressive disorder who were with high insomnia; MDDLI: Patients with major depressive disorder who were with low insomnia.

Abbreviation: HC: Healthy controls.

* *p* < 0.05.

For ReHo, 5 clusters showed significant group differences among patients with MDDHI, MDDLI, and HCs (Figure 2 and Table 2), including bilateral angular gyrus/precuneus/cerebellum posterior lobe/cuneus/lingual gyrus/middle temporal gyrus/middle occipital gyrus/limbic lobe/inferior parietal lobule, the left superior temporal gyrus, the left and right postcentral gyrus, and the right superior parietal lobule. Post hoc analyses showed that both patients with MDDHI and MDDLI exhibited decreased ReHo values compared to HCs in all clusters.

For DC, 5 clusters showed significant group differences among patients with MDDHI, MDDLI, and HCs (Figure 2 and Table 2), including the right superior temporal gyrus, the left lingual gyrus, the left and right precuneus, the left middle temporal gyrus and the left cuneus. Post hoc analyses showed that patients with MDDHI exhibited significantly decreased DC values compared to HCs in all five clusters. Patients with MDDLI showed numerically decreased DC values than HCs in all five clusters, but this difference reached statistical significance only in the right superior temporal gyrus, the left and right precuneus, and the left middle temporal gyrus (all *p* < 0.05), not in the left lingual gyrus (*p* = 0.229) or the left cuneus (*p* = 0.095).

### 3.4 Validation Analyses

For functional connectivity, when the global signal was not regressed, only the AG‐MFG connection survived, and patients with MDDLI again exhibited decreased functional connectivity than both patients with MDDHI and HCs. Moreover, 12 additional connections showed significant group differences among patients with MDDHI, MDDLI, and HCs. Details about these 12 connections could be found in Figure S2. When we excluded one site at a time, we found that the three significant connections (i.e., the AG‐MFG connection, the left and right middle frontal gyrus connection, and the left and right thalamus connection) remained significant (at a threshold of *p* < 0.001 per connection) when any single site was excluded, and the direction of the between‐group differences was consistent with our main findings (Figure S3 and Table S5). Moreover, all the other validation methods supported our main findings, with detailed results available in the Supporting Information.

For ALFF, ReHo and DC, the majority of local function abnormalities (i.e., decreases in patients vs. HCs) remained consistent when global signal was not regressed (Figure S4 and Table S6).

### 3.5 Correlation Analyses

**FIGURE 3:**
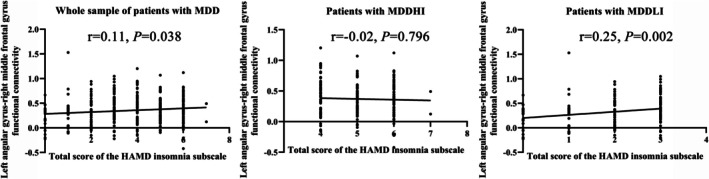
Correlation analyses between the AG‐MFG functional connectivity and the total score of the HAMD insomnia subscale.

The results showed that the AG‐MFG functional connectivity was positively correlated with the total score of the HAMD insomnia subscale in the whole sample of patients with MDD (*r* = 0.11, *p* = 0.038) and patients with MDDLI (*r* = 0.25, *p* = 0.002), but not patients with MDDHI (r = −0.02, *p* = 0.796) (Figure 3).

## 4 Discussion

Utilizing multi‐center, large‐sample data, we investigated local and network‐level functional abnormalities associated with insomnia severity in MDD. Our key finding was that patients with MDDLI exhibited decreased AG–MFG functional connectivity compared to both patients with MDDHI and HCs. And this result was relatively robust. In contrast, abnormalities in local brain function (ALFF, ReHo, DC) were widespread and observed in both MDD groups relative to HCs, but did not differ between patients with MDDHI and MDDLI.

The association between reduced AG‐MFG functional connectivity and lower insomnia severity in MDD represents a novel finding. Functionally, the left angular gyrus is critical for self‐referential processing [33], and negative rumination [34], whereas the right middle frontal gyrus serves as a core node of the executive control network, mediating top‐down inhibitory regulation of arousal and attention [35, 36]. Functional coupling between these regions is thought to support high‐order cognitive control over internally generated thoughts [35], a process relevant to sleep–wake regulation. Both regions have been implicated in sleep‐related neuroimaging studies. For example, within the sleep deprivation group, significant clusters of activation during resting fixation period relative to task stimulus viewing included the left angular gyrus and the right middle frontal gyrus [37]. Patients with MDD who were with low sleep efficiency exhibited increased functional connectivity strength in the left angular gyrus relative to those with normal sleep efficiency group [38]. Brain areas in which functional connectivity was associated with depressive problems and their effects on sleep quality included the angular gyrus [14]. Additionally, the central histaminergic neuron system, which is responsible for regulating wakefulness and sleep‐awake cycle, had reduced binding potential values of [11C]‐doxepin in bilateral middle frontal gyrus of patients with MDD [39]. And there was a decline in glucose metabolism in the middle frontal gyrus after sleep deprivation, but after sleep recovery, glucose metabolism was increased in the middle frontal gyrus [40].

Our results link reduced AG‐MFG functional connectivity to lower insomnia severity within MDD. This pattern suggests that diminished information exchange between these two regions may be associated with milder sleep disturbance, possibly reflecting reduced propagation of rumination‐related cognitive hyperarousal into sustained wakefulness [41, 42]. By contrast, the relatively preserved functional connectivity in patients with MDDHI may reflect a failure to disengage executive control from rumination‐driven self‐referential processing. Rather than representing effective regulation, this sustained coupling may constitute a vicious cycle in which the executive control network (i.e., the right middle frontal gyrus) is continuously recruited to monitor or suppress—rather than resolve—negative self‐referential thoughts originating from the left angular gyrus, thereby perpetuating cognitive hyperarousal. This interpretation aligns with the hyperarousal theory of insomnia [41, 42], which posits that persistent neurophysiological hyperarousal serves as both a core mechanism of insomnia and a neurobiological bridge linking insomnia and depression. Importantly, our results do not support a simple linear interpretation whereby AG‐MFG connectivity abnormality directly indexes greater insomnia severity. Rather, they point toward neurobiological heterogeneity in sleep‐related symptom profiles within MDD. Two potential interpretations are proposed: first, this connectivity may differentiate depressive subtypes characterized by distinct balances among arousal regulation, executive control, and self‐referential processing; second, it may reflect state‐dependent neural reorganization accompanying severe insomnia, with MDDLI representing an earlier stage where compensatory decoupling has not yet been engaged. However, these interpretations require further validation. Taken together, our findings advance understanding of the neural circuitry underlying insomnia in depression and highlight this connectivity as a candidate neuroimaging marker for sleep‐related heterogeneity in MDD, as well as a potential target for neuromodulation therapies aimed at improving sleep outcomes. Future longitudinal studies with repeated sleep assessments and treatment follow‐up are warranted to determine whether this connectivity serves as a stable trait marker, a state‐dependent indicator, or a predictor of therapeutic response.

We found that the AG‐MFG connectivity was positively correlated with the total score of the HAMD insomnia subscale in the whole sample of patients with MDD and the MDDLI subgroup. This aligns with our group comparison findings, indicating that reduced information exchange between these regions is associated with milder insomnia. The absence of a significant correlation in the MDDHI subgroup suggests that the relationship between this circuit and insomnia severity may be nonlinear or bounded, with this connectivity being informative primarily within the mild‐to‐moderate insomnia range. Whether this reflects a ceiling effect or a shift to alternative neural mechanisms in the severe subgroup requires further investigation.

Utilizing ALFF, ReHo and DC, our study revealed that abnormalities in local brain function were predominantly observed between patients with MDD and HCs, rather than between MDDHI and MDDLI subgroups. Affected regions included the angular gyrus, the precuneus, the middle temporal gyrus, the lingual gyrus and so on. Notably, while ALFF and ReHo consistently showed significant reductions in both patients with MDDHI and MDDLI compared to HCs, DC exhibited a more nuanced pattern with significant reductions in patients with MDDHI across all clusters and in patients with MDDLI in a subset of clusters. The majority of these regions are key components of the default mode network, the visuospatial processing network, and the executive control network, supporting self‐referential processing [33] and negative rumination [34]. The absence of subgroup differences suggests that reduced local spontaneous activity across MDD reflects core depressive pathology rather than sleep‐state‐dependent variation. However, whether these local activity reductions represent trait‐like vulnerabilities or state‐dependent concomitants of depression requires longitudinal validation. By contrast, the abnormal AG–MFG functional connectivity may represent a state‐dependent network reorganization that tracks insomnia severity. We hypothesize that reduced coupling between these regions may reflect diminished regulatory dialogue between self‐referential processing and executive control, potentially contributing to sleep–wake dysregulation. Our results thus add a novel dimension to the literature by moving beyond regional abnormalities to a network‐based perspective that differentiates shared depressive pathology from insomnia‐related circuitry. Moreover, we searched previous literature investigating insomnia‐related brain functional abnormalities in MDD using ALFF, ReHo and DC. For ALFF, only one prior small‐sample study (MDDHI:MDDLI = 24:37) reported that patients with MDDHI exhibited increased ALFF in the right inferior frontal gyrus/anterior insula, relative to MDDLI [18]; these findings await independent replication. For ReHo, two small‐sample studies (ratios of 22:22 and 24:33) yielded inconsistent findings [20, 21]. Our study, with a substantially larger sample (MDDHI:MDDLI = 226:159), did not replicate these subgroup differences in ALFF or ReHo. Several methodological factors may contribute to the discrepancy. First, small samples are vulnerable to sampling variability in clinical profiles, medication status, and comorbidity patterns, which can obscure stable group effects. Second, single‐center designs may further introduce site‐specific scanning parameters and population characteristics that reduce generalizability. Our large, carefully characterized multicenter cohort aims to mitigate these confounds, providing greater statistical power and enhancing confidence in the reproducibility of our negative findings. For DC, to our knowledge, no prior study has compared patients with MDDHI and MDDLIs; our MDD vs. HCs findings align with previous researches [43, 44]. Overall, the distributed local activity abnormalities observed in patients with MDD may contribute to a neuroimaging signature of depression‐related neural dysregulation. This not only enhances our understanding of the pathological mechanisms underlying MDD but also suggests that these abnormal brain function indicators could serve as potential biomarkers for clinical diagnosis.

The current study makes three specific contributions to understanding the neural mechanisms of insomnia in MDD. First, to our knowledge, it represents the largest multicenter study to date simultaneously examining local brain function and distributed functional connectivity in patients with MDD stratified by insomnia severity. Second, by showing that local abnormalities are common to MDD regardless of insomnia status whereas AG‐MFG functional connectivity differentiates subgroups, it helps clarify the neurobiological boundary between depression‐related deficits and insomnia‐related network alterations. Third, the multicenter design with standardized protocols offers greater confidence in the reproducibility of these findings, which may partly account for the inconsistency observed in earlier small‐sample studies. Taken together, these contributions advance the field by providing a more nuanced framework for understanding the neural substrates of sleep disturbance in MDD.

## 5 Limitations and Future Directions

Several limitations warrant consideration. First, although re‐running our second‐level analyses either with medication status included as a covariate or restricted to unmedicated patients yielded consistent support for our primary findings (See Supporting Information), we still cannot rule out potential medication‐related effects on brain function. The consistency of our local function findings with prior studies [43, 44] suggests the core functional connectivity finding related to insomnia severity might also be relatively robust. Second, despite standardized preprocessing protocols and statistical control, MRI acquisition parameters varied across sites; possible site‐related variance in neuroimaging metrics may still exist. However, after harmonizing our functional connectivity matrices using the ComBat method, the results continue to support our main findings (See Supporting Information). Third, the cross‐sectional design precludes causal inferences about the temporal relationship between the AG–MFG functional connectivity changes and insomnia trajectory. Fourth, the sample was restricted to Chinese adults within a specific age range, limiting generalizability to other ethnicities, age groups, or cultural contexts. Fifth, the use of the HAMD insomnia items for both patient classification and ROC analysis represents a form of self‐validation. The resulting cutoff score (≥ 4) should therefore be considered preliminary and requires external validation. Second, we did not employ dedicated sleep questionnaires such as the Pittsburgh Sleep Quality Index (PSQI) or the Insomnia Severity Index (ISI), which would have provided independent and more comprehensive assessments of sleep disturbance.

These limitations directly inform specific future research directions that build upon our core findings. First, future studies should ideally recruit drug‐naïve patients or employ a structured washout/wash‐in protocol with repeated scanning. This would disentangle whether observed connectivity patterns are medication artifacts, depression‐state correlates, or insomnia‐specific mechanisms. Second, future multicenter studies should adopt harmonized scanning protocols and include diverse ethnic and age cohorts. Extending the current findings to adolescent and late‐life depression populations is especially important given developmental and aging‐related changes in default mode network–executive control network dynamics and sleep architecture. Third, prospective longitudinal studies should track the AG–MFG connectivity before, during, and after evidence‐based insomnia intervention in patients with MDD, such as cognitive behavioral therapy for insomnia [CBT‐I], pharmacological treatment, or transcranial magnetic stimulation (TMS). This would clarify whether (a) baseline AG–MFG connectivity predicts treatment response, (b) successful insomnia remission is accompanied by normalization or further reduction of this connectivity, and (c) connectivity changes precede or follow clinical improvement. Fourth, independent external cohorts are needed to validate the HAMD insomnia cutoff threshold of ≥ 4 identified in the current work. Standardized sleep questionnaires such as PSQI and ISI should be incorporated to achieve more objective and comprehensive assessments of sleep symptoms and cross‐validate the brain functional correlates of insomnia in mood disorders.

## 6 Conclusion

This large‐scale, multi‐center study identified a specific reduction in functional connectivity between the left angular gyrus and the right middle frontal gyrus that was associated with lower insomnia severity in patients with MDD. In contrast, widespread reductions in local brain function (ALFF, ReHo, DC) appear to be characterstic of MDD broadly, independent of insomnia severity. These results significantly advance our understanding of the distinct neurobiological substrates underlying insomnia symptoms within MDD versus the core pathophysiology of the disorder itself. The identified left angular gyrus—right middle frontal gyrus connection represents a novel and promising target for developing interventions specifically aimed at alleviating insomnia in individuals with MDD.

## Author Contributions

Wang CY and Bo QJ designed the study and wrote the protocol. Zhang ZF managed the formal analysis, investigation, methodology and project administration. Chen ZZ, Wang YM, Zhao L, Li F, Xing YJ and Li T managed the data curation. Zhang ZF and Bo QJ undertook the statistical analysis. Zhang ZF wrote the first draft of the manuscript. Wang CY and Bo QJ reviewed and edited the manuscript.

## Funding

This work was supported by the Brain Science and Brain‐like Intelligence Technology‐National Science and Technology Major Project (2021ZD0200600) and the Beijing Hospitals Authority Clinical Medicine Development of special funding support (ZLRK202335) and Capital Medical University (PYZ25178) and Capital Health Development Scientific Research Special Project (Shoufa 2024‐2G‐1172).

## Ethics Statement

This study was conducted in accordance with the Declaration of Helsinki and approved by each site's ethics committee the Ethics Committee (2008) Clinical Review No. (14). All participants provided written informed consent prior to enrollment.

## Conflicts of Interest

The authors declare no conflicts of interest.

## Supporting information


**Figure S1:** Flow chart of participant inclusion and exclusion.
**Figure S2:** Results of functional connectivity analyses among patients with major depressive disorder who were with high/low insomnia and healthy controls when the global signal was not regressed out.
**Figure S3:** Results of functional connectivity analyses among patients with major depressive disorder who were with high/low insomnia and healthy controls when one of the nine site was left out.
**Figure S4:** Results of the ALFF, ReHo and DC analyses among patients with major depressive disorder who were with high/low insomnia and healthy controls when the global signal was not regressed out.
**Table S1:** Contributing sample size and key MRI acquisition parameters for each site.
**Table S2:** Demographic characteristics and group differences stratified by site.
**Table S3:** Site differences in HAMD total scores.
**Table S4:** Internal Consistency of the HAMD Insomnia Subscale by Site.
**Table S5:** The result distribution of each center in the leave‐one‐out validation analyses.
**Table S6:** Results of the ALFF, ReHo and DC analyses among patients with major depressive disorder who were with high/low insomnia and healthy controls when the global signal was not regressed out.

## Data Availability

The data that support the findings of this study are available from the Depression Imaging REsearch ConsorTium (DIRECT) REST‐meta‐MDD Project at http://rfmri.org/REST‐meta‐MDD. The data are openly shared through the Psychological Science Data Bank (https://doi.org/10.57760/sciencedb.o00115.00013).
