# Supplementary material for: Association of Left Angular Gyrus‐Right Middle Frontal Gyrus Functional Connectivity With Insomnia Severity in Major Depressive Disorder: Evidence From the DIRECT Consortium
Source: CNS Neurosci Ther. 2026 Aug 13;32(8):e71083. doi: 10.1002/cns.71083 (PMC13472150; doi:10.1002/cns.71083)
Supplement: Supplementary file 1 [file CNS-32-e71083-s001.pdf]

SUPPLEMENTARY MATERIALS

Content

Figure S1. Flow chart of participant inclusion and exclusion. 2
Figure S2. Results of functional connectivity analyses among patients with major depressive disorder who were with high/low insomnia and healthy controls when the global signal was not regressed out. 3
Figure S3. Results of functional connectivity analyses among patients with major depressive disorder who were with high/low insomnia and healthy controls when one of the nine site was left out. 4
Figure S4. Results of the ALFF, ReHo and DC analyses among patients with major depressive disorder who were with high/low insomnia and healthy controls when the global signal was not regressed out. 5
Table S1. Contributing sample size and key MRI acquisition parameters for each site. 6
Table S2. Demographic characteristics and group differences stratified by site. 7
Table S3. Site differences in HAMD total scores...9
Table S4. Internal Consistency of the HAMD Insomnia Subscale by Site. 11
Table S5. The result distribution of each center in the leave-one-out validation analyses. 12
Table S6. Results of the ALFF, ReHo and DC analyses among patients with major depressive disorder who were with high/low insomnia and healthy controls when the global signal was not regressed out. 15
ROC Analysis for Insomnia Subscale Threshold Validation...18
Validation analysis: ComBat harmonization...20
Validation analysis: medication status was included as an additional covariate............................22
Validation analysis: only recruited unmedicated patients with MDD 23
Validation analysis: partial correlation analysis controlling for non-sleep depressive symptoms ..24
Multiple regression analysis...25

Figure S1. Flow chart of participant inclusion and exclusion.

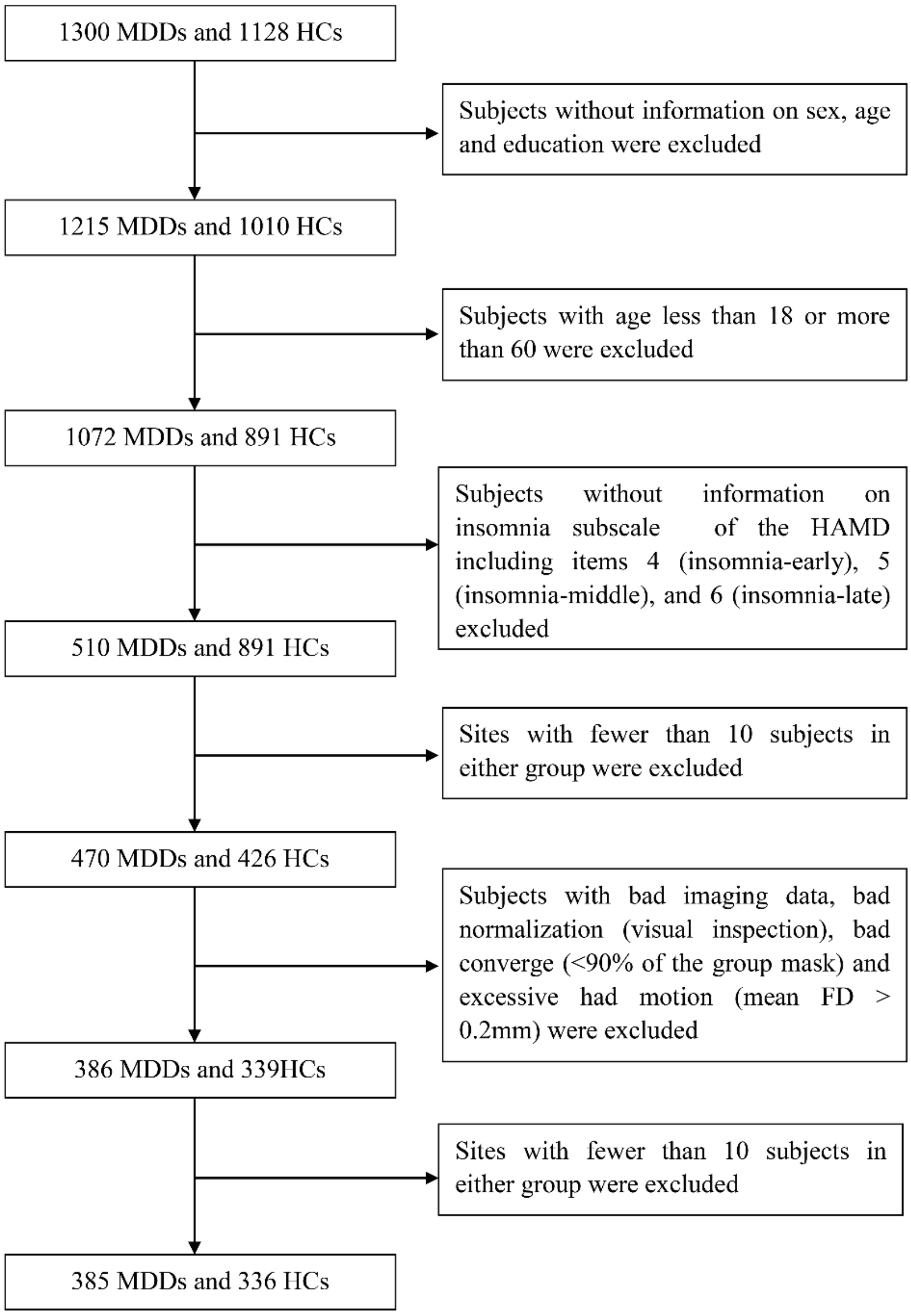

Figure S2. Results of functional connectivity analyses among patients with major depressive disorder who were with high/low insomnia and healthy controls when the global signal was not regressed out.

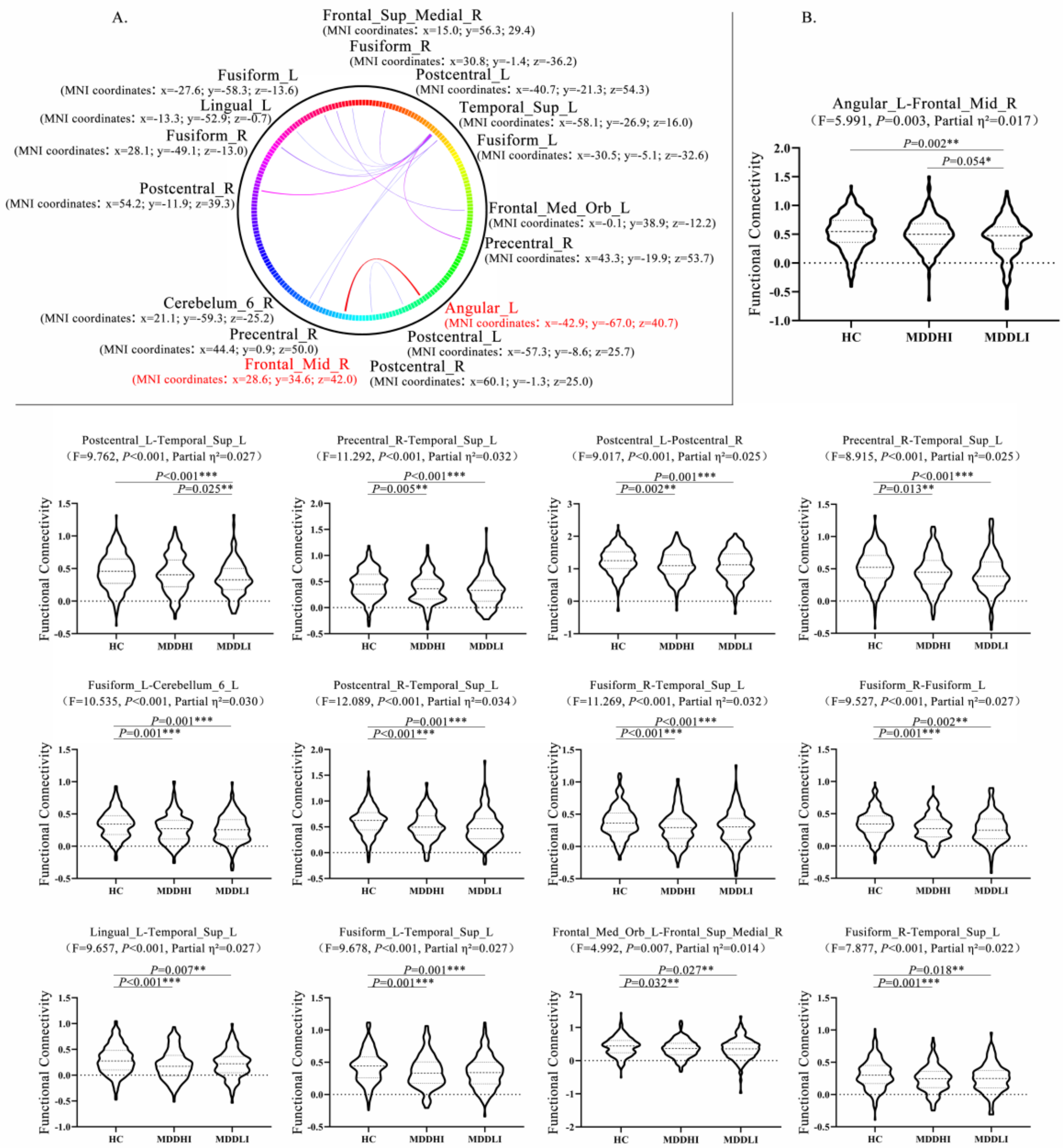

Note: **P*≤0.1, *P*≤0.05,**P*≤0.001.

Figure S3. Results of functional connectivity analyses among patients with major depressive disorder who were with high/low insomnia and healthy controls when one of the nine site was left out.

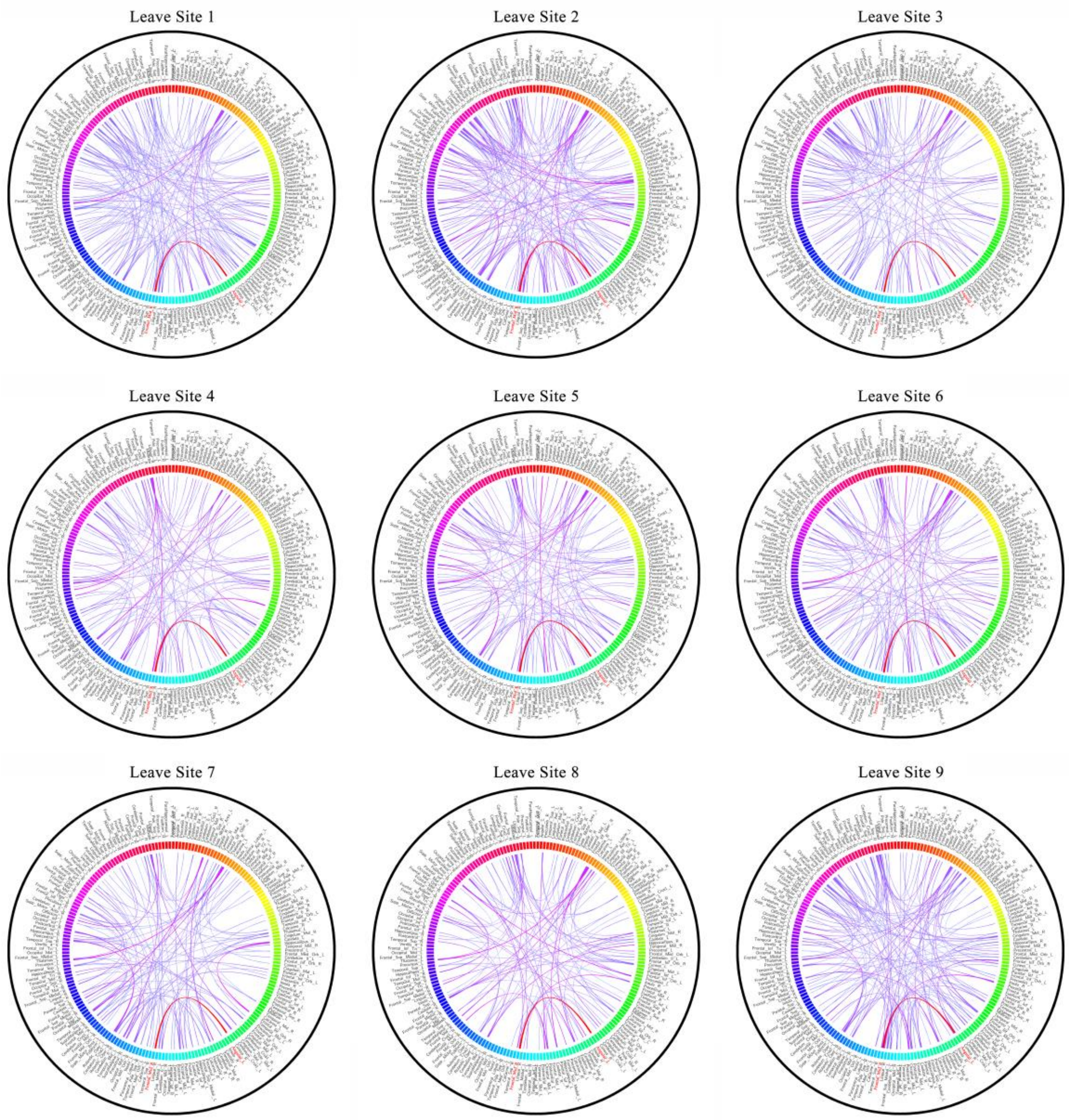

Figure S4. Results of the ALFF, ReHo and DC analyses among patients with major depressive disorder who were with high/low insomnia and healthy controls when the global signal was not regressed out.

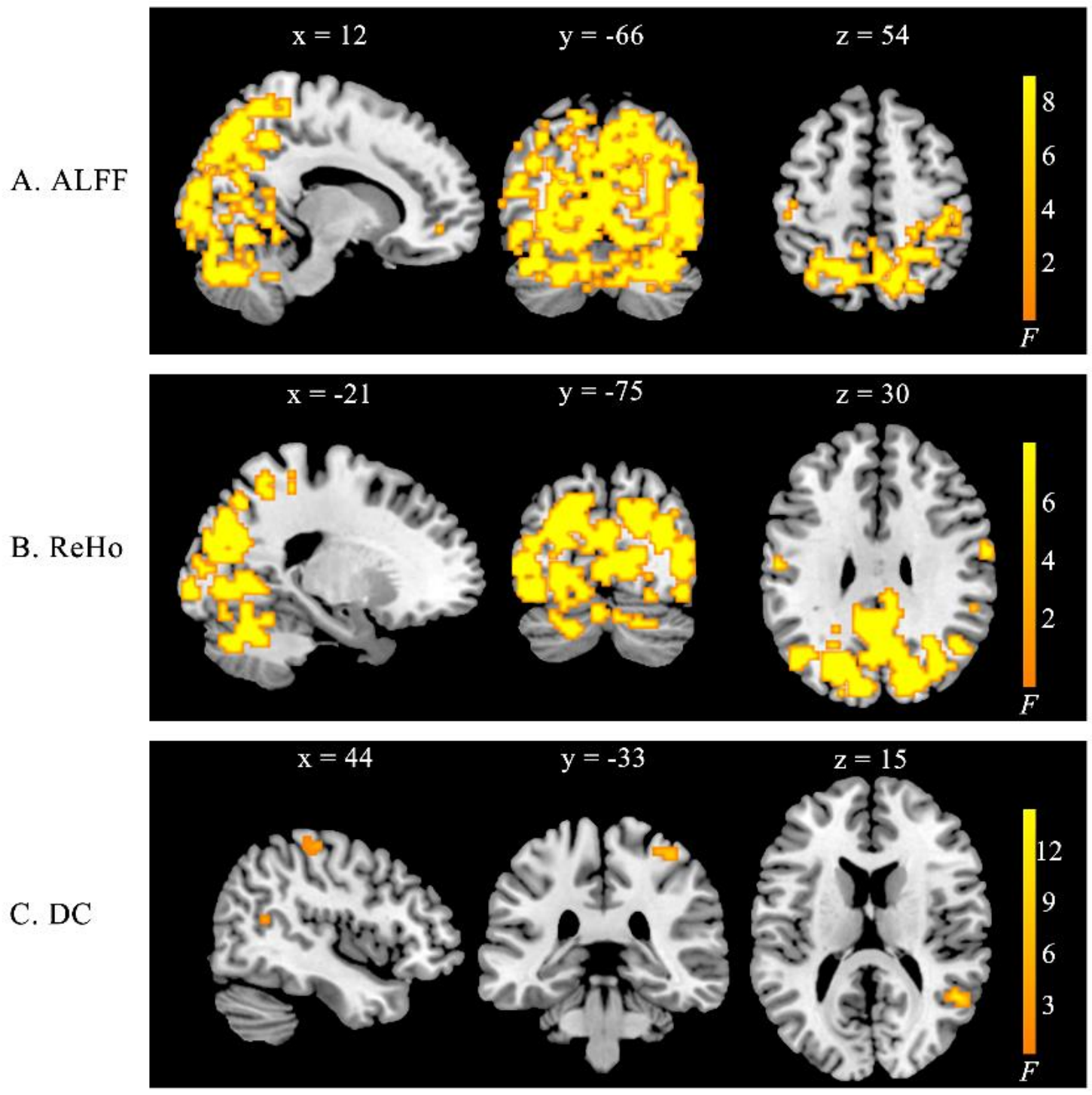

Table S1. Contributing sample size and key MRI acquisition parameters for each site.

Site	Samples		MR Scanner	TR(ms)	TE(ms)	Time points
	MDD	HC				
1	45	40	Siemens Tim Trio 3T	2000	30	210
2	25	20	Philips Achieva 3T	2000	30	200
3	34	38	GE discovery MR750	2000	30	184
4	56	62	GE Signa 3T	2000	30	200
5	61	32	Siemens Magnetom Symphony scanner 1.5 T	2000	40	150
6	40	31	GE Signa 3T	2000	22.5	240
7	77	65	Siemens Tim Trio 3T	2000	30	240
8	23	20	Philips Gyroscan Acheva 3.0T	2000	30	250
9	24	28	Philips Achieva 3.0T TX	2000	30	240

Note: MDD: major depressive disorder; HC: healthy control; TR: repetition time; TE: echo time. The nine sites were respectively located at: 1) Peking University Sixth Hospital; 2) Suzhou Psychiatric Hospital; 3) Sir Run Run Shaw Hospital, Zhejiang University School of Medicine; 4) Department of Psychiatry, First Affiliated Hospital, China Medical University; 5) The Second Xiangya Hospital of Central South University; 6) Anhui Medical University; 7) Beijing Anding Hospital, Capital Medical University; 8) The Institute of Mental Health, Second Xiangya Hospital of Central South University; 9) Mental Health Center, West China Hospital, Sichuan University.

Table S2. Demographic characteristics and group differences stratified by site.

Site	Characteristics	MDD	HC	Z / χ^2(P)
1	Age[a]	30(26,36)	27(24,33.80)	-1.41(0.160)
	Gender (male/female)[b]	24/21	23/17	0.15(0.700)
	Education[a]	15(13,15)	15(15,15)	-2.49(0.013*)
	mean FD[a]	0.10(0.07,0.12)	0.10(0.07,0.12)	-0.31(0.758)
	HAMD	25(19.50,28)		
2	Age[a]	41(31.50,52.50)	49(34.80,53)	-0.80(0.424)
	Gender (male/female)[b]	3/22	6/14	2.25(0.134)
	Education[a]	11(8,15)	8(5.75,14.50)	-0.60(0.552)
	mean FD[a]	0.06(0.04,0.09)	0.07(0.05,0.12)	-1.71(0.087)
	HAMD	23(21,24.50)		
3	Age[a]	40(31,51)	33.5(29,47.30)	-1.33(0.185)
	Gender (male/female)[b]	13/21	15/23	0.12(0.914)
	Education[a]	10.50 (6.75, 15)	16(12,17.50)	-4.04(<0.001*)
	mean FD[a]	0.05(0.04,0.07)	0.05(0.04,0.07)	-0.27(0.787)
	HAMD	22(19,23)		
4	Age[a]	31(24.50,41)	28(22,39)	-0.83(0.408)
	Gender (male/female)[b]	19/37	26/36	0.80(0.371)
	Education[a]	11(8,14.50)	13(11,15)	-2.97(0.003*)
	mean FD[a]	0.05(0.03,0.06)	0.05(0.04,0.07)	-0.15(0.884)
	HAMD	24(21,31)		
5	Age[a]	29(25,34.50)	28(27,32.80)	-0.15(0.881)
	Gender (male/female)[b]	19/42	15/17	2.24(0.135)
	Education[a]	14(12,16)	15(15,16)	-1.34(0.182)
	mean FD[a]	0.04(0.03,0.05)	0.04(0.03,0.05)	-0.66(0.507)
	HAMD	21(19,23)		
6	Age[a]	37(25,45)	38(27,44)	-0.59(0.558)
	Gender (male/female)[b]	14/26	17/14	2.79(0.095)
	Education[a]	8(8,11)	11(8,15)	-1.37(0.171)
	mean FD[a]	0.05(0.04,0.07)	0.04(0.03,0.06)	-1.98(0.047*)
	HAMD	23(17.50,27)		
7	Age[a]	30(24,42.50)	36(23.50,47.50)	-1.47(0.142)
	Gender (male/female)[b]	34/43	28/37	0.02(0.897)
	Education[a]	13(11,15)	13(11,15)	-2.52(0.012*)
	mean FD[a]	0.06(0.04,0.10)	0.07(0.05,0.10)	-1.08(0.280)

	HAMD	14(7.50,20)		
8	Age[a]	31(26,43)	21(20,25.30)	-2.97(0.003*)
	Gender (male/female)[b]	10/13	12/8	1.17(0.280)
	Education[a]	12(9,13)	13.50(13,15)	-2.16(0.031*)
	mean FD[a]	0.07(0.05,0.11)	0.07(0.05,0.09)	-0.58(0.559)
	HAMD	22(19,26)		
9	Age[a]	24.5(20.30,26)	26.5(24,45.80)	-2.51(0.012*)
	Gender (male/female)[b]	14/10	11/17	1.88(0.171)
	Education[a]	15(9.75,16)	17(9,17)	-1.89(0.058)
	mean FD[a]	0.07(0.06,0.08)	0.07(0.06,0.09)	-0.26(0.797)
	HAMD	20(18,25.75)		

Note: MDD: major depressive disorder; HC: healthy control; FD: Framewise displacement. The nine sites were respectively located at: 1) Peking University Sixth Hospital; 2) Suzhou Psychiatric Hospital; 3) Sir Run Run Shaw Hospital, Zhejiang University School of Medicine; 4) Department of Psychiatry, First Affiliated Hospital, China Medical University; 5) The Second Xiangya Hospital of Central South University; 6) Anhui Medical University; 7) Beijing Anding Hospital, Capital Medical University; 8) The Institute of Mental Health, Second Xiangya Hospital of Central South University; 9) Mental Health Center, West China Hospital, Sichuan University.

[a]This variable was compared by using Mann-Whitney U test;

[b]This variable was compared by using χ^2 test;

$^{*}P < 0.05$

Table S3. Site differences in HAMD total scores.

Site	Site	HAMD total scores	
		t	*p*
1	2	-0.758	1.000
	3	-1.967	1.000
	4	-0.124	1.000
	5	-3.269	0.039*
	6	-1.693	1.000
	7	-7.071	0.000
	8	-1.107	1.000
	9	-2.425	0.552
2	3	-0.979	1.000
	4	-0.682	1.000
	5	-1.909	1.000
	6	-0.702	1.000
	7	-4.943	<0.001*
	8	-0.328	1.000
	9	-1.483	1.000
3	4	-1.941	1.000
	5	-0.913	1.000
	6	-0.339	1.000
	7	-4.273	0.001*
	8	-0.604	1.000
	9	-0.622	1.000
4	5	-3.336	0.031*
	6	-1.657	1.000
	7	-7.413	<0.001*
	8	-1.046	1.000
	9	-2.410	0.575
5	6	-1.349	1.000
	7	-3.993	0.002*
	8	-1.465	1.000
	9	-0.122	1.000
6	7	-4.920	<0.001*
	8	-0.321	1.000
	9	-0.948	1.000
7	8	-4.389	<0.001*
	9	-3.054	0.081
8	9	-1.127	1.000

Note: HAMD: Hamilton depression scale. The nine sites were respectively located at: 1) Peking University Sixth Hospital; 2) Suzhou Psychiatric Hospital; 3) Sir Run Run Shaw Hospital, Zhejiang University School of Medicine; 4) Department of Psychiatry, First Affiliated Hospital, China Medical University; 5) The Second Xiangya Hospital of Central South University; 6) Anhui Medical University; 7) Beijing Anding Hospital, Capital Medical University; 8) The Institute of Mental Health, Second Xiangya Hospital of Central South University; 9) Mental Health Center,

West China Hospital, Sichuan University. *$P < 0.05$

Table S4. Internal Consistency of the HAMD Insomnia Subscale by Site.

Site	n	MDDHI	MDDLI	Mean	Standard deviation	Cronbach's α
1	45	27	18	3.8	1.36	0.236
2	61	43	18	4.23	1.42	0.194
3	40	30	10	4.8	2.03	0.851
4	25	19	6	4.36	0.95	0.243
5	77	24	53	2.49	1.85	0.606
6	23	12	11	3.43	1.93	0.581
7	24	12	12	3.79	1.59	0.561
8	34	23	11	4.29	1.53	0.752
9	56	36	20	4.14	1.77	0.602
total	385	226	159	3.84	1.75	0.626

Note: MDDHI: Patients with major depressive disorder who were with high insomnia; MDDLI: Patients with major depressive disorder who were with low insomnia.

Table S5. The result distribution of each center in the leave-one-out validation analyses.

Connections	F	*P*	partial η^2	Post-hoc (Bonferroni correction)
Leave Site 1				
The left and right middle frontal cortex connection	15.861	<0.001*	0.050	HC>MDDHI, *P*=0.048*<br>HC>MDDLI, *P*<0.001*<br>MDDHI>MDDLI, *P*=0.007*
The left angular gyrus - the right middle frontal cortex connection	10.501	<0.001*	0.033	HC>MDDHI, *P*=0.811<br>HC>MDDLI, *P*<0.001*<br>**MDDHI>MDDLI, *P*=0.004***
The left and right thalamus connection	17.342	<0.001*	0.054	HC>MDDHI, *P*<0.001*<br>HC>MDDLI, *P*<0.001*<br>MDDHI>MDDLI, *P*=1.000
Leave Site 2				
The left and right middle frontal cortex connection	15.324	<0.001*	0.045	HC>MDDHI, *P*=0.144<br>HC>MDDLI, *P*<0.001*<br>MDDHI>MDDLI, *P*=0.003*
The left angular gyrus - the right middle frontal cortex connection	9.379	<0.001*	0.028	HC>MDDHI, *P*=0.981<br>HC>MDDLI, *P*<0.001*<br>**MDDHI>MDDLI, *P*=0.007***
The left and right thalamus connection	13.261	<0.001*	0.039	HC>MDDHI, *P*<0.001*<br>HC>MDDLI, *P*<0.001*<br>MDDHI>MDDLI, *P*=1.000
Leave Site 3				
The left and right middle frontal cortex connection	10.718	<0.001*	0.033	HC>MDDHI, *P*=0.284<br>HC>MDDLI, *P*<0.001*<br>MDDHI>MDDLI, *P*=0.016*
The left angular gyrus - the right middle frontal cortex connection	6.614	0.001*	0.021	HC>MDDHI, *P*=1.000<br>HC>MDDLI, *P*=0.002*<br>**MDDHI>MDDLI, *P*=0.008***
The left and right thalamus connection	15.649	<0.001*	0.048	HC>MDDHI, *P*<0.001*<br>HC>MDDLI, *P*<0.001*<br>MDDHI>MDDLI, *P*=1.000
Leave Site 4				
The left and right middle frontal cortex connection	13.204	<0.001*	0.044	HC>MDDHI, *P*=0.037*<br>HC>MDDLI, *P*<0.001*<br>MDDHI>MDDLI, *P*=0.037*

The left angular gyrus - the right middle frontal cortex connection	7.626	0.001*	0.026	HC>MDDHI, *P*=0.716<br>HC>MDDLI, *P*<0.001*<br>**MDDHI>MDDLI, *P*=0.032***
The left and right thalamus connection	11.589	<0.001*	0.039	HC>MDDHI, *P*<0.001*<br>HC>MDDLI, *P*<0.001*<br>MDDHI>MDDLI, *P*=1.000
Leave Site 5				
The left and right middle frontal cortex connection	14.021	<0.001*	0.045	HC>MDDHI, *P*=0.329<br>HC>MDDLI, *P*<0.001*<br>MDDHI>MDDLI, *P*=0.003*
The left angular gyrus - the right middle frontal cortex connection	10.882	<0.001*	0.035	HC>MDDHI, *P*=1.000<br>HC>MDDLI, *P*<0.001*<br>**MDDHI>MDDLI, *P*=0.001***
The left and right thalamus connection	14.047	<0.001*	0.045	HC>MDDHI, *P*<0.001*<br>HC>MDDLI, *P*<0.001*<br>MDDHI>MDDLI, *P*=1.000
Leave Site 6				
The left and right middle frontal cortex connection	12.120	<0.001*	0.036	HC>MDDHI, *P*=0.136<br>HC>MDDLI, *P*<0.001*<br>MDDHI>MDDLI, *P*=0.015*
The left angular gyrus - the right middle frontal cortex connection	8.843	<0.001*	0.027	HC>MDDHI, *P*=1.000<br>HC>MDDLI, *P*<0.001*<br>**MDDHI>MDDLI, *P*=0.006***
The left and right thalamus connection	12.242	<0.001*	0.037	HC>MDDHI, *P*<0.001*<br>HC>MDDLI, *P*=0.001*<br>MDDHI>MDDLI, *P*=1.000
Leave Site 7				
The left and right middle frontal cortex connection	8.232	<0.001*	0.029	HC>MDDHI, *P*=0.032*<br>HC>MDDLI, *P*<0.001*<br>MDDHI>MDDLI, *P*=0.337
The left angular gyrus - the right middle frontal cortex connection	6.654	0.001*	0.024	HC>MDDHI, *P*=1.000<br>HC>MDDLI, *P*=0.001*<br>**MDDHI>MDDLI, *P*=0.010***
The left and right thalamus connection	15.534	<0.001*	0.053	HC>MDDHI, *P*<0.001*<br>HC>MDDLI, *P*=0.020*<br>MDDHI<MDDLI, *P*=0.262
Leave Site 8				

The left and right middle frontal cortex connection	13.900	<0.001*	0.041	HC>MDDHI, *P*=0.091<br>HC>MDDLI, *P*<0.001*<br>MDDHI>MDDLI, *P*=0.009*
The left angular gyrus - the right middle frontal cortex connection	7.825	<0.001*	0.024	HC>MDDHI, *P*=1.000<br>HC>MDDLI, *P*<0.001*<br>**MDDHI>MDDLI, *P*=0.011***
The left and right thalamus connection	13.797	<0.001*	0.041	HC>MDDHI, *P*<0.001*<br>HC>MDDLI, *P*<0.001*<br>MDDHI>MDDLI, *P*=1.000
Leave Site 9				
The left and right middle frontal cortex connection	15.755	<0.001*	0.047	HC>MDDHI, *P*=0.022*<br>HC>MDDLI, *P*<0.001*<br>MDDHI>MDDLI, *P*=0.013*
The left angular gyrus - the right middle frontal cortex connection	11.276	<0.001*	0.034	HC>MDDHI, *P*=0.353<br>HC>MDDLI, *P*<0.001*<br>**MDDHI>MDDLI, *P*=0.007***
The left and right thalamus connection	12.079	<0.001*	0.036	HC>MDDHI, *P*<0.001*<br>HC>MDDLI, *P*=0.001*<br>MDDHI>MDDLI, *P*=1.000

Note: $^{*}P<0.05$

Table S6. Results of the ALFF, ReHo and DC analyses among patients with major depressive disorder who were with high/low insomnia and healthy controls when the global signal was not regressed out.

	Brain region	Hemisphere	MNI coordinates	Cluster range coordinates	Peak F values	Cluster size	Cluster-level P_{FWE}	Post-hoc analysis	
								Comparisons	$P_{Bonferroni}$
ALFF	Angular/Precuneus/Cerebellum Posterior Lobe/Cuneus/Lingual Gyrus/Middle Temporal Gyrus/Middle Occipital Gyrus/Limbic Lobe	Left/Right	0,-63,15	X: -63 to 66<br>Y: -99 to -18<br>Z: -39 to 69	23.38	7106	<0.001*	MDDHI < MDDLI<br>MDDHI < HC<br>MDDLI < HC	1.000<br>< 0.001*<br>< 0.001*
	Medial Frontal Gyrus	Left/Right	3,57,21	X: -12 to 12<br>Y: 21 to 63<br>Z: -18 to 33	16.68	212	<0.001*	MDDHI < MDDLI<br>MDDHI < HC<br>MDDLI < HC	1.000<br>< 0.001*<br>< 0.001*
			3,36,48	X: -3 to 6<br>Y: 27 to 45<br>Z: 36 to 51	11.68	30	0.001*	MDDHI < MDDLI<br>MDDHI < HC<br>MDDLI < HC	1.000<br>< 0.001*<br>0.001*
	Superior Frontal Gyrus	Left	-21,54,30	X: -33 to -9<br>Y: 21 to 54<br>Z: 15 to 36	15.97	54	<0.001*	MDDHI < MDDLI<br>MDDHI < HC<br>MDDLI < HC	1.000<br>< 0.001*<br>0.001*
			-21,33,45	X: -24 to -18<br>Y: 18 to 33<br>Z: 42 to 48	12.37	17	0.041*	MDDHI < MDDLI<br>MDDHI < HC<br>MDDLI < HC	1.000<br>< 0.001*<br>< 0.001*
	Inferior Parietal Lobule	Left	-45,-30,45	X: -54 to -39<br>Y: -33 to -12<br>Z: 36 to 54	16.87	45	<0.001*	MDDHI < MDDLI<br>MDDHI < HC<br>MDDLI < HC	1.000<br>0.001*<br>0.001*
			-54,-27,30	X: -63 to -48	14.89	19	0.023*	MDDHI < MDDLI	1.000

			Y: -39 to -27				MDDHI < HC	< 0.001*
			Z: 24 to 33				MDDLI < HC	< 0.001*
			X: -39 to -15				MDDHI < MDDLI	1.000
Middle Frontal Gyrus	Left	-21,63,12	Y: 51 to 66	15.62	56	<0.001*	MDDHI < HC	< 0.001*
			Z: -3 to 18				MDDLI < HC	0.001*
			X: -51 to -36				MDDHI < MDDLI	1.000
		-45,18,-6	Y: 15 to 27	12.07	19	0.023*	MDDHI < HC	0.001*
			Z: -12 to 0				MDDLI < HC	0.001*
	Left							
			X: -57 to -39				MDDHI < MDDLI	1.000
Inferior Frontal Gyrus		-45,24,15	Y: 21 to 33	12.38	20	0.018*	MDDHI < HC	< 0.001*
			Z: 9 to 24				MDDLI < HC	0.002*
			X: 33 to 48				MDDHI < MDDLI	1.000
	Right	45,24,-15	Y: 15 to 30	11.35	22	0.011*	MDDHI < HC	< 0.001*
			Z: -21 to -12				MDDLI < HC	0.001*
			X: -45 to -33				MDDHI < MDDLI	1.000
	Left	-39,24,-21	Y: 15 to 33	13.71	25	0.005*	MDDHI < HC	< 0.001*
			Z: -27 to -6				MDDLI < HC	0.001*
Superior Temporal Gyrus								
			X: 48 to 66				MDDHI < MDDLI	1.000
	Right	63,-21,6	Y: -27 to -9	12.96	39	<0.001*	MDDHI < HC	0.001*
			Z: -6 to 9				MDDLI < HC	< 0.001*
			X: -66 to -57				MDDHI < MDDLI	1.000
Middle Temporal Gyrus	Left	-60,-24,-12	Y: -36 to -18	10.60	20	0.018*	MDDHI < HC	0.002*
			Z: -18 to -3				MDDLI < HC	0.002*
			X: -63 to -51				MDDHI < MDDLI	1.000
Postcentral Gyrus	Left	-54,-3,39	Y: -21 to -3	11.02	32	0.001*	MDDHI < HC	0.001*
			Z: 24 to 39				MDDLI < HC	0.001*

ReHo	Angular/Precuneus/Cuneus/Limbic Lobe/Cerebellum Posterior Lobe/Middle Occipital Gyrus/Middle Temporal Gyrus/Lingual Gyrus/Inferior Parietal Lobule	Left/Right	-21,-75,30	X: -63 to 60<br>Y: -102 to -15<br>Z: -39 to 69	21.51	5655	<0.001*	MDDHI < MDDLI<br>MDDHI < HC<br>MDDLI < HC	1.000<br>< 0.001*<br>< 0.001*
	Superior Temporal Gyrus	Left	-60,-18,0	X: -66 to -48<br>Y: -27 to -9<br>Z: -6 to 39	14.69	141	<0.001*	MDDHI < MDDLI<br>MDDHI < HC<br>MDDLI < HC	1.000<br>< 0.001*<br>< 0.001*
	Postcentral Gyrus	Right	63,-6,27	X: 57 to 63<br>Y: -12 to 3<br>Z: 12 to 30	14.05	34	0.007*	MDDHI < MDDLI<br>MDDHI < HC<br>MDDLI < HC	1.000<br>< 0.001*<br>0.002*
	Middle Temporal Gyrus	Left	-57,-27,-12	X: -63 to -54<br>Y: -30 to -18<br>Z: -18 to -9	14.09	30	0.013*	MDDHI < MDDLI<br>MDDHI < HC<br>MDDLI < HC	1.000<br>< 0.001*<br>0.001*
DC	Superior Temporal Gyrus	Right	51,-54,15	X: 45 to 54<br>Y: -63 to -51<br>Z: 15 to 27	13.77	16	0.026*	MDDHI < MDDLI<br>MDDHI < HC<br>MDDLI < HC	1.000<br>< 0.001*<br>0.005*
	Postcentral Gyrus	Right	39,-27,54	X: 30 to 45<br>Y: -36 to -24<br>Z: 54 to 63	10.45	17	0.019*	MDDHI < MDDLI<br>MDDHI < HC<br>MDDLI < HC	1.000<br>< 0.001*<br>0.022*

Note: MDDHI: Patients with major depressive disorder who were with high insomnia; MDDLI: Patients with major depressive disorder who were with low insomnia; HC: Healthy controls; *$P < 0.05$

ROC Analysis for Insomnia Subscale Threshold Validation

To empirically evaluate the validity of the a priori grouping threshold for insomnia severity (i.e. MDDLI ≤3 vs. MDDHI ≥4 on the HAMD insomnia subscale), we conducted a receiver operating characteristic (ROC) curve analysis (Hanley et al., 1982). Given the absence of an independent insomnia-specific instrument in this study, we adopted an internal criterion based on the HAMD-17 sleep items themselves: a patient was classified as having clinically significant insomnia if any of the three sleep items (early, middle, or late insomnia) was rated ≥2 (moderate or above). This criterion reflects the clinical convention that a single moderate-to-severe sleep disturbance warrants clinical attention. The HAMD insomnia subscale total score (0–6) served as the test variable.

The ROC analysis yielded an AUC of 95.7% (95% CI: 94.0%–97.4%, $P < 0.001$). See figure bellow:

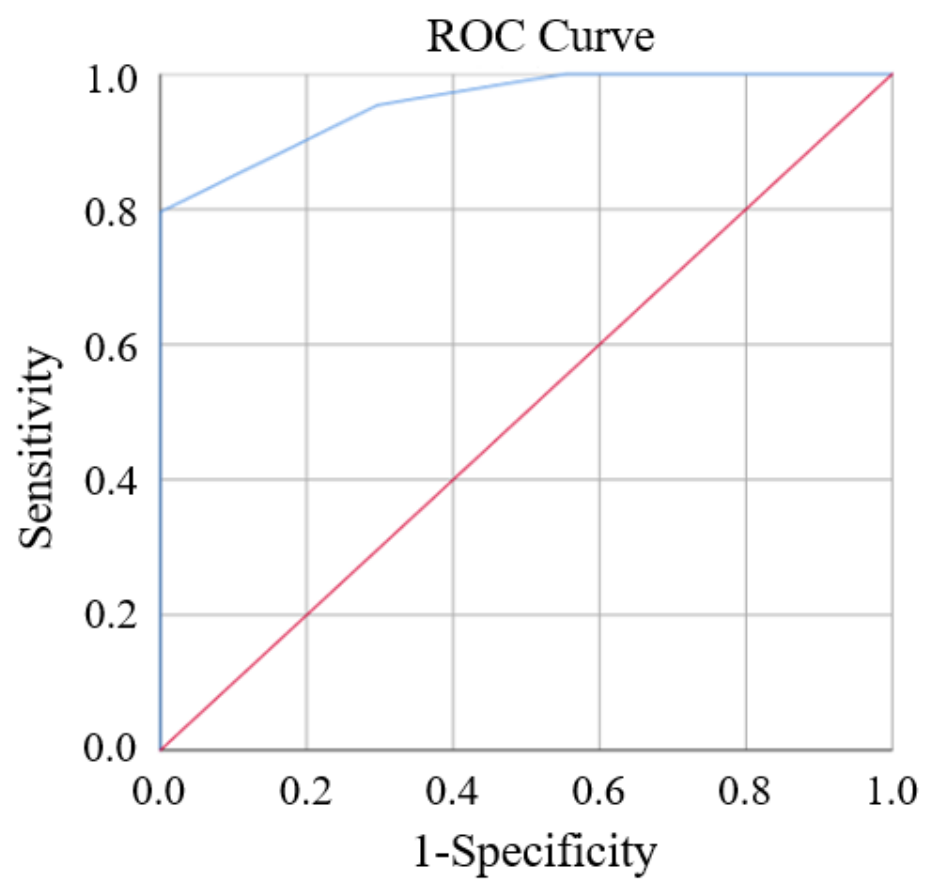

At the threshold of 3.5 (corresponding to our predefined a priori cut-off of ≥4), the sensitivity was 79.6% and specificity 100.0%. The full coordinate points read as follows:

Positive when greater than or equal to this value	Sensitivity	1-Specificity
-1.00	1.000	1.000
0.50	1.000	0.762
1.50	1.000	0.554
2.50	0.954	0.297
**3.50**	**0.796**	**0.000**
4.50	0.535	0.000
5.50	0.282	0.000
6.50	0.021	0.000
7.50	0.011	0.000
8.50	0.004	0.000

10.00	0.000	0.000

The near-perfect specificity (100%) is mathematically expected: by definition, a patient with all three sleep items rated ≤1 (mild or absent) cannot achieve a total score ≥4, as the maximum possible total under this condition is 3. Consequently, the ≥4 threshold and the "any item ≥2" criterion are structurally aligned rather than independent. The high AUC therefore reflects the internal coherence of the HAMD sleep items rather than external diagnostic validity against an independent gold standard.

The sensitivity of 79.6% indicates that the ≥4 threshold successfully identifies approximately four-fifths of patients with at least one moderate-to-severe sleep symptom, while the 20.4% false-negative rate primarily captures patients with a single moderate symptom accompanied by minimal disturbance in other domains (e.g., scores of 2+0+0 = 2, or 2+1+0 = 3). These cases represent milder or more focal insomnia phenotypes that fall below the multi-symptom threshold of ≥4.

The ≥4 threshold thus operationalizes a conservative but clinically meaningful construct: it identifies patients with either multi-domain mild insomnia or single-domain moderate-to-severe insomnia with additional mild involvement (e.g., 2+1+1 = 4). This aligns with the clinical concept of "high insomnia burden" requiring targeted intervention, as opposed to patients with isolated mild symptoms who may represent a distinct phenotypic subgroup.

We fully acknowledge that this ROC analysis relies on an internal criterion derived from the same instrument, rather than an independent gold standard (e.g., PSQI, ISI, or polysomnography). The high AUC is therefore best interpreted as evidence of structural validity within the HAMD-17 framework. The justification for the ≥4 threshold rests on three converging lines of evidence: ①Structural validity (present ROC analysis): The threshold aligns with the internal clinical-significance criterion of any sleep item ≥2. ②Literature precedent: The ≥4 cut-off has been widely adopted in prior neuroimaging and clinical studies of MDD-related insomnia (Epperson et al., 2023; Liu et al., 2018; Ye et al., 2023). ③Sensitivity-priority principle: For neuroimaging analyses, sensitivity in capturing the phenotype of interest is prioritized over perfect specificity, ensuring adequate enrichment of the MDDHI subgroup.

Validation analysis: ComBat harmonization

Using all participants' functional connectivity matrices, ComBat harmonization was performed using the DPABI built-in implementation (Wang et a., 2024). Here, scanning site was defined as the batch variable, with age, sex, education level, and mean FD included as covariates. Diagnosis (patients with MDDHI vs. patients with MDDLI vs. HCs) was specified as the biological variable of interest to preserve disease-related variance and avoid premature removal of true group differences. Principal component analysis (PCA) was performed on the upper triangular elements of the whole brain functional connectivity matrix to visualize site related variance.

The results showed that, before harmonization, some separation among sites could be observed along the first two principal components. After ComBat harmonization, the site-specific clusters were effectively dissipated, with all site distributions converging toward a common centroid and showing substantial overlap. Notably, the total variance explained by the first two principal components remained nearly unchanged (PCA1: 4.0% → 3.9%; PCA2: 3.0% → 2.9%), indicating that ComBat selectively removed site-related variance without substantially altering the overall data structure or compressing genuine biological variance. See figure below:

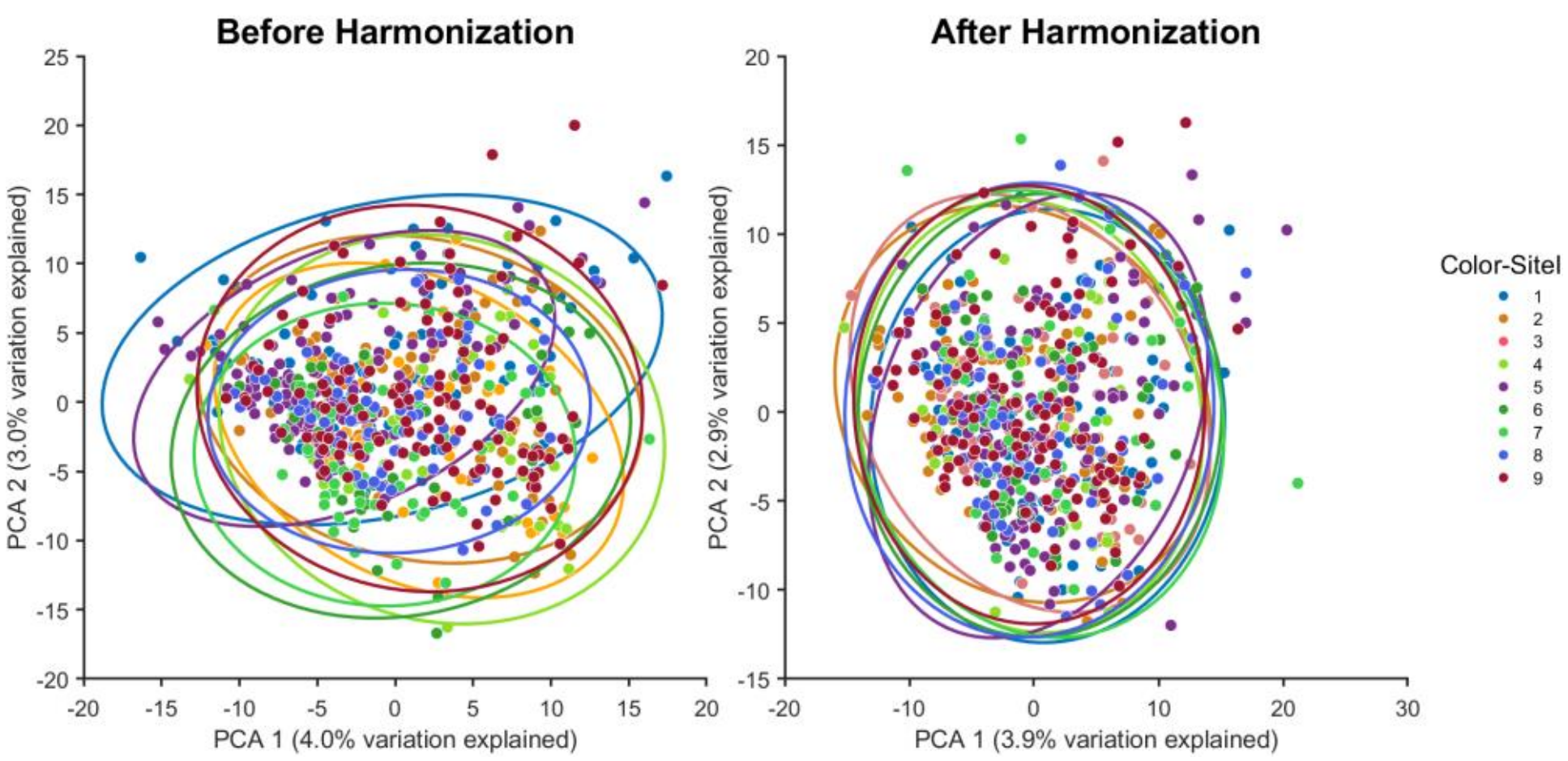

Then we re-conducted the group-level ANCOVA on the harmonized data, with diagnostic group (patients with MDDHI vs. patients with MDDLI vs. HCs) as the independent variable and age, sex, education level, and mean FD as covariates. While the overall number of FDR-corrected significant connections decreased — consistent with previous reports that data-level harmonization can conservatively attenuate both site-related and some disease-related variance in

multi-site functional connectivity data (Habes et al., 2021) — the left angular gyrus-right middle frontal gyrus connection (our primary finding) remained among the top-ranked connections by uncorrected significance (P = 1.1581e-05). Moreover, post-hoc analysis showed that patients with MDDLI exhibited decreased functional connectivity than both patients with MDDHI and HCs (all $P_{\text{Bonferroni-corrected}}<0.05$). See figure below:

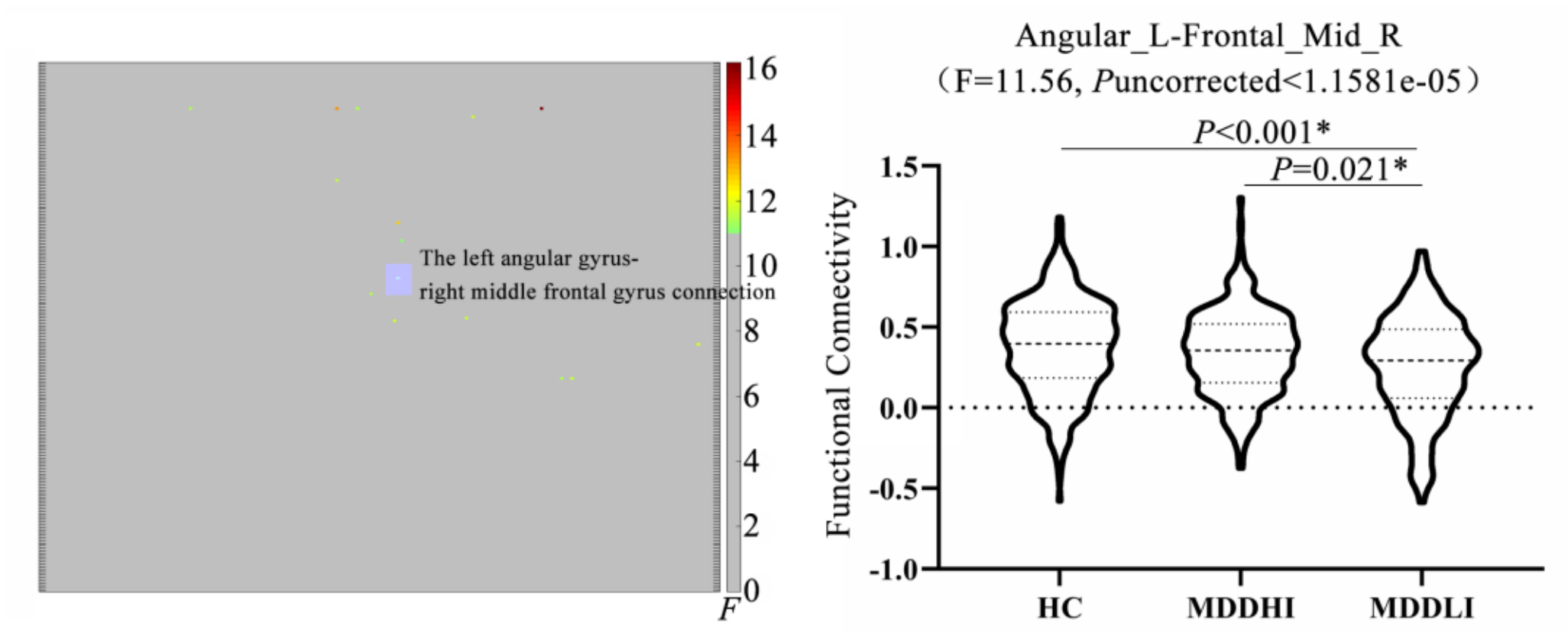

The persistence of this connection as a key group-differentiating feature under both site-covariate regression and ComBat harmonization provides convergent evidence that the observed result is unlikely to be entirely driven by site-related artifacts.

Validation analysis: medication status was included as an additional covariate

In addition to the covariates of age, gender, education level, mean FD, and scanning site, medication status (unmedicated, medicated, or unknown) was also included as an additional covariate in the second-level analysis of the functional connectivity. The results showed that the AG-MFG connection also exhibited significant group differences among patients with MDDHI, MDDLI and HCs ($F=10.73$, $P_{FDR\text{-}corrected}<0.05$). Post-hoc analysis showed that patients with MDDLI exhibited decreased functional connectivity than both patients with MDDHI and HCs (all $P_{Bonferroni\text{-}corrected}<0.05$). See figure below:

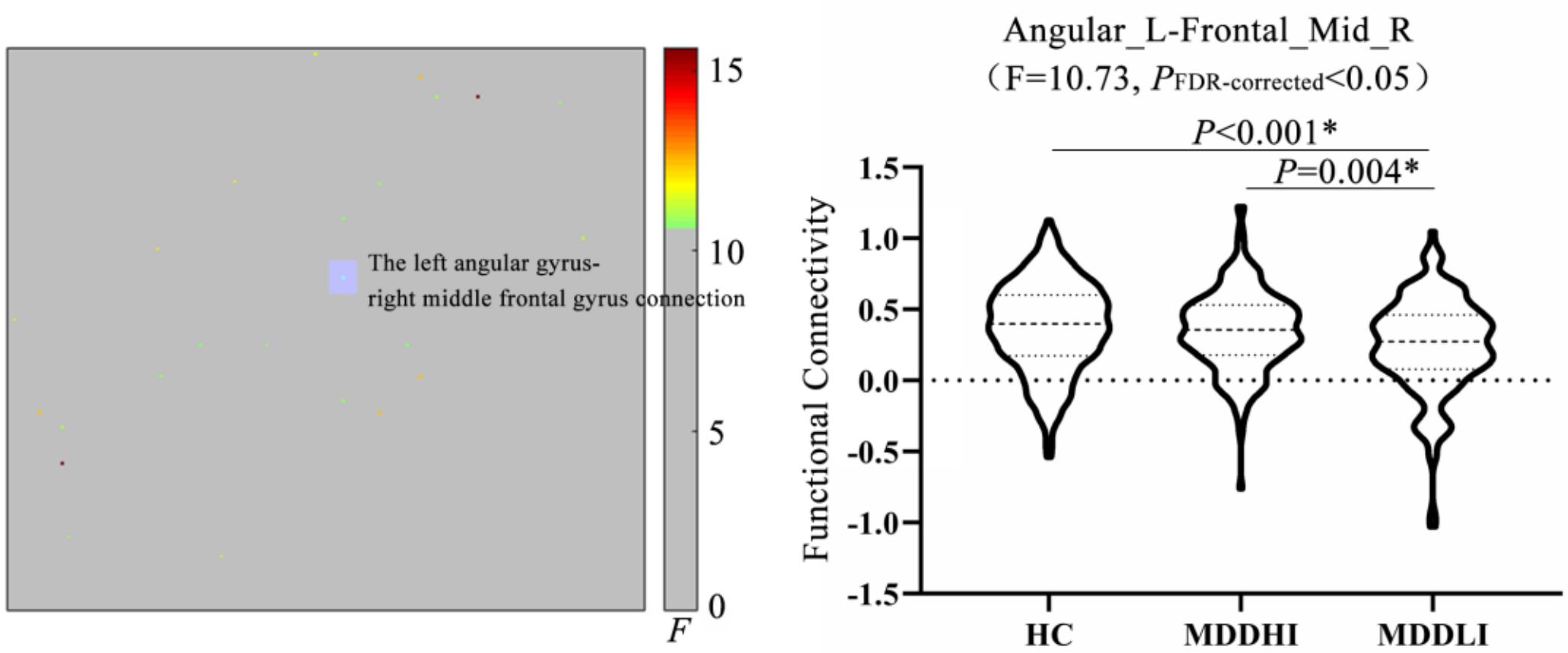

Validation analysis: only recruited unmedicated patients with MDD

Based on our statistical summary, there were 87 unmedicated patients in the MDDHI group and 49 in the MDDLI group. To mitigate site-related bias, we retained only sites with more than 10 patients in both groups. Consequently, 2 sites were included in each group, yielding final sample sizes of 69 (MDDHI) and 34 (MDDLI). A total of 94 HCs from these two sites were also included.

Then, we re-performed a one-way ANCOVA analysis in DPABI software. In this analysis, group (patients with MDDHI vs. patients with MDDLI vs. HCs) was independent variable, whole-brain functional connectivity matrix was dependent variable, and age, gender, education level, head motion, and site were covariates. Unfortunately, no edge passed the FDR correction. However, after extracting the functional connectivity values of the left angular gyrus–right middle frontal gyrus connection for each subject, we found that the pattern of high and low values across the three subject groups was consistent with the main results reported in our main manuscript (i.e. HCs > MDDHI > MDDLI), as illustrated in the figure below:

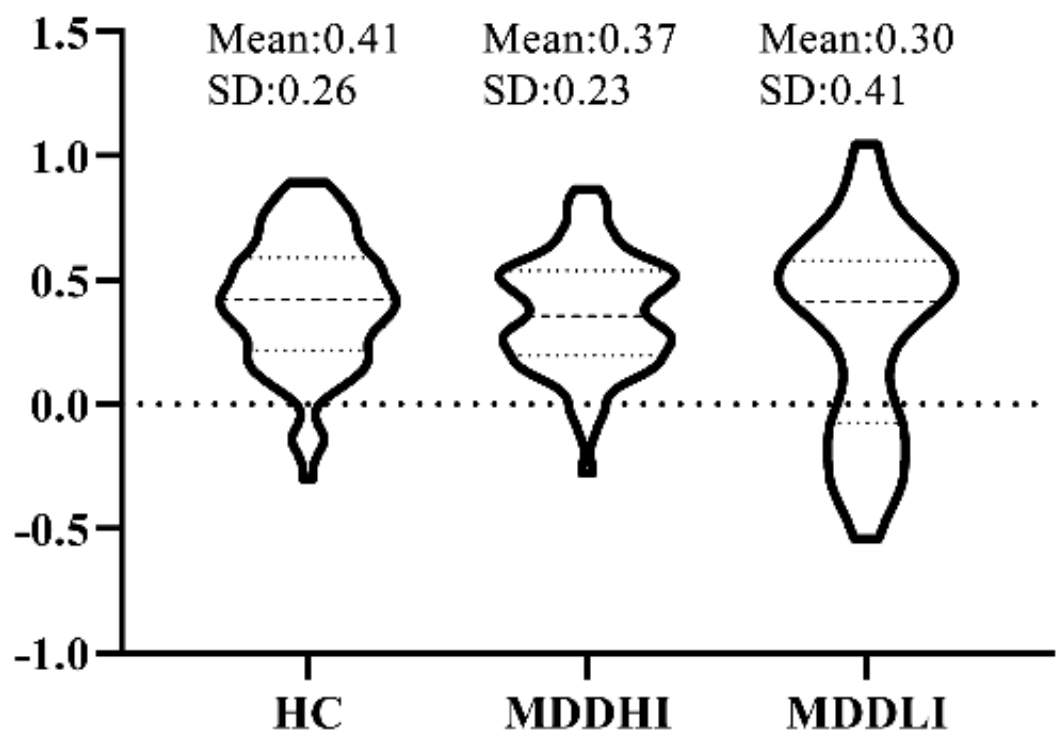

However, we emphasize that this result cannot be interpreted as demonstrating medication independence. Being unmedicated at baseline only indicates no medication use at enrollment, and participants with a history of medication exposure prior to scanning cannot be excluded. Moreover, 32% of the sample had unknown status, and medication use is associated with insomnia severity, rendering covariate adjustment susceptible to measurement bias and overadjustment. These analyses are best understood as showing that a crude enrollment-timepoint medication variable does not fully capture the observed group differences—a finding that is consistent with, but not proof of, disease-driven rather than medication-driven effects.

Validation analysis: partial correlation analysis controlling for non-sleep depressive symptoms

In SPSS 26.0 software, we conducted a partial correlation analysis between AG-MFG connectivity and HAMD insomnia subscale score (items 4+5+6), controlling for adjusted HAMD total score (i.e. total HAMD score minus the insomnia subscale), age, gender, education, mean FD, and scanning site, restricted to the patient sample (MDDHI and MDDLI) (Cohen et al., 2003; Field, 2013). The partial correlation was statistically significant but modest in magnitude ($r = 0.109$, $P = 0.043$, two-tailed), suggesting that AG-MFG connectivity retains some association with insomnia severity independent of non-sleep depressive symptoms. However, we interpret this result with caution for several reasons:

First, the effect size is small ($r^2 \approx 0.012$), explaining approximately 1.2% of the variance in AG-MFG connectivity after controlling for covariates. Second, HAMD insomnia and non-insomnia scores are inherently correlated in clinical samples, and controlling for both simultaneously may inflate standard errors (Vatcheva et al., 2016). Third, the HAMD insomnia subscale (items 4+5+6) captures only nocturnal symptoms and is a coarse measure of insomnia severity compared to dedicated instruments such as the ISI.

Multiple regression analysis

To more rigorously examine whether insomnia severity independently predicts AG-MFG connectivity, by using the SPSS 26.0 software, we conducted a multiple linear regression analysis in the combined patient sample (MDDHI and MDDLI) (Slinker et al., 2008). AG-MFG connectivity was entered as the dependent variable, HAMD insomnia subscale score (items 4+5+6) as the primary continuous predictor, and the adjusted HAMD total score (i.e. HAMD non-insomnia score), age, gender, education, mean FD, and scanning site as covariates. The overall model was significant (F = 2.90, *P* = 0.001, adjusted R^2 = 0.069). Crucially, HAMD insomnia subscale score significantly predicted AG-MFG connectivity (β = 0.121, *P* = 0.043), explaining an additional 1.1% of variance beyond the covariates (ΔR^2 = 0.011, ΔF = 4.14, *P* = 0.043). The variance inflation factor for HAMD insomnia score was 1.369, indicating no serious multicollinearity with HAMD non-insomnia score or other covariates. This result converges with our partial correlation analysis (r = 0.109, *P* = 0.043), providing consistent evidence that insomnia severity is associated with AG-MFG connectivity independent of non-sleep depressive symptoms. However, we interpret this result with caution, because the effect size is modest, and the explained variance is small (ΔR^2 = 0.011).

Reference:

Hanley JA, McNeil BJ. The meaning and use of the area under a receiver operating characteristic (ROC) curve. Radiology. 1982;143(1):29-36.

Epperson CN, Rubinow DR, Meltzer-Brody S, et al. Effect of brexanolone on depressive symptoms, anxiety, and insomnia in women with postpartum depression: Pooled analyses from 3 double-blind, randomized, placebo-controlled clinical trials in the HUMMINGBIRD clinical program. J Affect Disord. 2023;320:353-359.

Liu CH, Guo J, Lu SL, et al. Increased Salience Network Activity in Patients With Insomnia Complaints in Major Depressive Disorder. Front Psychiatry. 2018;9:93.

Ye Y, Wang C, Lan X, et al. Abnormal amygdala functional connectivity in MDD patients with insomnia complaints. Psychiatry Res Neuroimaging. 2023;328:111578.

Wang YW, Wang HL, Yan CG. DPABI harmonization: A toolbox for harmonizing multi-site brain imaging for big-data era. Imaging Neurosci (Camb). 2024;2.

Habes M, Pomponio R, Shou H, et al. The Brain Chart of Aging: Machine-learning analytics reveals links between brain aging, white matter disease, amyloid burden, and cognition in the iSTAGING consortium of 10,216 harmonized MR scans. Alzheimers Dement. 2021;17(1):89-102.

Cohen J, Cohen P, West SG, et al. Applied multiple regression/correlation analysis for the behavioral sciences (3rd ed.). Lawrence Erlbaum Associates. 2003.

Field A. Discovering statistics using IBM SPSS statistics (4th ed.). SAGE Publications. 2013.

Vatcheva KP, Lee M, McCormick JB, Rahbar MH. Multicollinearity in Regression Analyses Conducted in Epidemiologic Studies. Epidemiology (Sunnyvale). 2016;6(2).

Slinker BK, Glantz SA. Multiple linear regression: accounting for multiple simultaneous determinants of a continuous dependent variable. Circulation. 2008;117(13):1732-1737.
